# The Association Between Vitamin C and Cancer: A Two-Sample Mendelian Randomization Study

**DOI:** 10.3389/fgene.2022.868408

**Published:** 2022-05-05

**Authors:** Hanxiao Chen, Ze Du, Yaoyao Zhang, Mengling Li, Rui Gao, Lang Qin, Hongjing Wang

**Affiliations:** ^1^ Department of Obstetrics and Gynaecology, West China Second University Hospital, Sichuan University, Chengdu, China; ^2^ Key Laboratory of Birth Defects and Related Diseases of Women and Children of the Ministry of Education, West China Second University Hospital, Sichuan University, Chengdu, China; ^3^ Reproductive Center, Department of Obstetrics and Gynaecology, West China Second University Hospital, Sichuan University, Chengdu, China; ^4^ Department of Orthopedics, Research Institute of Orthopedics, West China Hospital/West China School of Medicine, Sichuan University, Chengdu, China

**Keywords:** vitamin c, cancer, GWAS, SNP, Mendelian randomization

## Abstract

In recent years, many studies have indicated that vitamin C might be negatively associated with the risk of cancer, but the actual relationship between vitamin C and cancer remains ambivalent. Therefore, we utilized a two-sample Mendelian randomization (MR) study to explore the causal associations of genetically predicted vitamin C with the risk of a variety of cancers. Single-nucleotide polymorphisms (SNPs) associated with vitamin C at a significance level of *p* < 5 × 10–8 and with a low level of linkage disequilibrium (LD) (r2 < 0.01) were selected from a genome-wide association study (GWAS) meta-analysis of plasmid concentration of vitamin C consisting of 52,018 individuals. The data of the GWAS outcomes were obtained from United Kingdom Biobank, FinnGen Biobank and the datasets of corresponding consortia. In the inverse-variance weight (IVW) method, our results did not support the causal association of genetically predicted vitamin C with the risk of overall cancer and 14 specific types of cancer. Similar results were observed in sensitivity analyses where the weighted median and MR-Egger methods were adopted, and heterogeneity and pleiotropy were not observed in statistical models. Therefore, our study suggested that vitamin C was not causally associated with the risk of cancer. Further studies are warranted to discover the potential protective and therapeutic effects of vitamin C on cancer, and its underlying mechanisms.

## Introduction

Vitamin C, also called ascorbic acid, is a water-soluble vitamin commonly considered an electron donor with an antioxidant function that can eliminate fatal reactive oxygen species (ROS) (Lane and Richardson, 2014). On the other hand, vitamin C can also be a pro-oxidant at a pharmacological plasma concentration (Padayatty and Levine, 2016). In recent years, many researchers have indicated that vitamin C might be negatively associated with the risk of cancer (Bo et al., 2016; Aune et al., 2018; Jenkins et al., 2021), but the actual relationship and the underlying mechanisms of vitamin C in the pathogenesis or therapeutic effect of cancer remain ambivalent.

Cancer is the second-leading cause of death in the USA and causes approximately 600,000 deaths each year (Islami et al., 2020). Thus, prevention and treatment of cancer are of vital importance. Although cancer is known to be associated with some genetic and environmental factors and different cancers may have different risk factors, some studies suggested that vitamin C may also influence the development of cancer. However, previous studies have yielded inconclusive findings on the potential impact of vitamin C on cancer. One systematic review and dose–response meta-analysis study revealed that when the concentration of vitamin C in blood increased to 50 μmol/L, the relative risk (RR) for total cancer risk was 0.74 (95% confidence interval (CI): 0.66–0.82) (Aune et al., 2018). On the other hand, another systematic review that included 19 trials did not support the positive effect of vitamin C supplementation in patients with cancer on their clinical status and overall survival (van Gorkom et al., 2019). In addition, the relationship between vitamin C and cancer risk may be different in different types of cancer. Vitamin C has been linked to a lower risk of renal cell carcinoma, esophageal cancer, colon cancer, breast cancer, endometrial cancer, and cervical cancer (Bandera et al., 2009; Park et al., 2010; Fulan et al., 2011; Jia et al., 2015a; Bo et al., 2016). However, some studies also suggested that supplementary intake of vitamin C had no relationship with the risk of pancreatic cancer, bladder cancer, prostate cancer, cervical cancer, and ovarian cancer (Jiang et al., 2010; Chen et al., 2015; Cao et al., 2016; Hua et al., 2016; Long et al., 2020). Therefore, the causal role of vitamin C in the development of cancers remains unclear and warrants future studies.

A Mendelian randomization (MR) study uses genetic variation, typically single-nucleotide polymorphisms (SNPs), associated with an exposure to assess its potential causal relationship with an outcome. Compared with traditional observational studies, the MR study provides relatively more convincing evidence for detecting the association between the exposure and the outcome. The MR study can minimize the potential bias generated by potential confounding factors and reverse causality and will not be affected by disease progression because the genetic variants that are used as instrument variables (IVs) in the MR study are strongly and solely related to the exposure (Little, 2018). Using two-sample MR analysis, many studies have found a potential relationship between many risk factors and the risk of cancer (Larsson et al., 2020; Yuan et al., 2020). However, the causal association between vitamin C and the risk of cancer has not yet been fully established using MR analysis. A recent MR study did not support the association between vitamin C and five types of cancer, including lung, breast, prostate, colon, and rectal cancer (Fu et al., 2021), but whether there are causal associations between vitamin C and other types of cancer remains unclear.

Therefore, in this study, we aimed to comprehensively explore the causal associations of genetically predicted vitamin C with the risk of different types of cancer by utilizing a two-sample MR study.

## Materials and Methods

### Study Design

**FIGURE 1:**
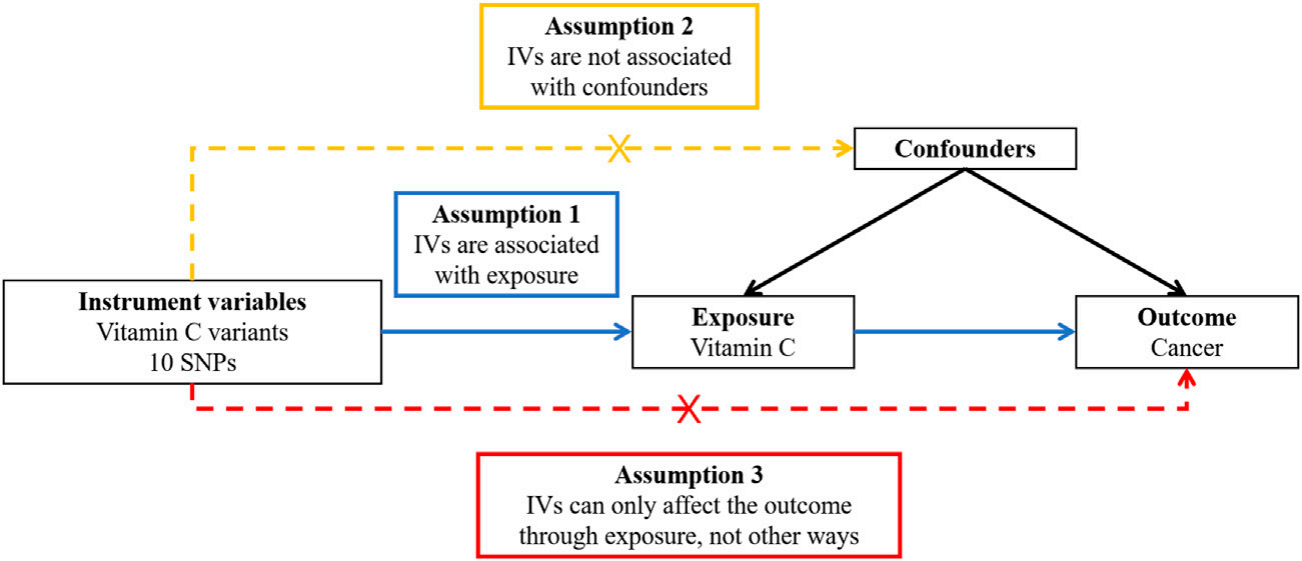
Overview of the design and three key assumptions of the Mendelian randomization study. IVs, instrument variables; SNPs, single-nucleotide polymorphisms.

In order to obtain reliable results from a two-sample MR study, the genetic variants used in this study should be in conformity with three principles (Figure 1), including the relevance assumption, independence assumption, and exclusion restriction assumption, which means these genetic variants should be strongly related to the exposure (i.e., vitamin C), be not associated with confounding factors of the exposure–outcome relationship, and have an effect on the outcome (i.e., cancer) only through the exposure and not any other pathway (Little, 2018).

### Genetic Instrumental Variables for Vitamin C

The SNPs associated with vitamin C were selected from a genome-wide association study (GWAS) meta-analysis of vitamin C (Zheng et al., 2021) consisting of 52,018 individuals from the following studies: 10,771 participants from the Fenland study (Ashor et al., 2017); 16,841 participants from the European Prospective Investigation into Cancer and Nutrition (EPIC)-InterAct study (Consortium, 2011); 16,756 participants from the EPIC Norfolk study (Day et al., 1999) (excluding duplicated samples with EPIC-InterAct); and 7,650 participants from the EPIC-CVD study (Danesh et al., 2007) (excluding duplicated samples with EPIC-InterAct or EPIC-Norfolk). A total of 11 independent SNPs were reported to be related to vitamin C at the genome-wide significance level (*p* < 5 × 10^−8^). Since rs7740812 was correlated (*r* ^2^ < 0.01) in linkage disequilibrium (LD) analysis, the remaining 10 SNPs were included to establish the genetic IVs for vitamin C (Table 1).

**TABLE 1:** Vitamin C SNPs used to construct the instrument variable.

Chr	Position	SNP	Effect allele	Other allele	EAF	Beta	SE	Gene	*p* Value	F Statistics
1	2330190	rs6693447	T	G	0.551	0.039	0.006	RER1	6.25E-10	42.25
2	220031255	rs13028225	T	C	0.857	0.102	0.009	SLC23A3	2.38E-30	128.4444
5	138715502	rs33972313	C	T	0.968	0.36	0.018	SLC23A1	4.61E-90	400
5	176799992	rs10051765	C	T	0.342	0.039	0.007	RGS14	3.64E-09	31.04082
11	61570783	rs174547	C	T	0.328	0.036	0.007	FADS1	3.84E-08	26.44898
12	96249111	rs117885456	A	G	0.087	0.078	0.012	SNRPF	1.70E-11	42.25
12	102093459	rs2559850	A	G	0.598	0.058	0.006	CHPT1	6.30E-20	93.44444
14	105253581	rs10136000	A	G	0.283	0.04	0.007	AKT1	1.33E-08	32.65306
16	79740541	rs56738967	C	G	0.321	0.041	0.007	MAF	7.62E-10	34.30612
17	59456589	rs9895661	T	C	0.817	0.063	0.008	BCAS3	1.05E-14	62.01563

Abbreviations: Chr, chromosome; SNP, single-nucleotide polymorphism; EAF, effect allele frequency; SE, standard error.

### Genetic Association Datasets for Cancer

Overall cancer and ten types of site-specific cancer were included as cancer outcomes in our MR study (Table 2). GWAS summary statistics on overall cancer and nine site-specific cancers, including lung, breast, colon, rectum, kidney, bladder, prostate, ovarian, and uterine/endometrial cancer, were obtained from the United Kingdom Biobank dataset. Summary statistics of GWAS on overall cancer and malignant neoplasm of the bronchus and lung, breast, pancreas, colon, rectum, kidney, bladder, prostate, ovary, and corpus uteri were acquired from the FinnGen Biobank database. Summary statistics of GWAS on lung cancer were obtained from the International Lung Cancer Consortium (ILCCO) (Wang et al., 2014). Summary statistics of GWAS on breast cancer were obtained from the Breast Cancer Association Consortium (BCAC) (Michailidou et al., 2017). GWAS summary statistics on pancreatic cancer were obtained from the Pancreatic Cancer Cohort Consortium (PanScan1) (Amundadottir et al., 2009). The GWAS summary of prostate cancer was derived from the Prostate Cancer Association group to Investigate Cancer Associated Alterations in the Genome (PRACTICAL) (Schumacher et al., 2018). Summary statistics of GWAS on ovarian cancer were obtained from the Ovarian Cancer Association Consortium (OCAC) (Phelan et al., 2017). In this study, we extracted the effect estimates and standard errors for each of the 10 vitamin C–related SNPs from the meta-GWAS summary statistics of overall cancer risk and site-specific cancer risk.

**TABLE 2:** Characteristics of included studies or consortia of cancer.

Type of Cancer	Source	Year	Sample size	Population
Overall cancer	UKBB	2018	461311	European
	FinnGen Biobank	2020	96499	European
Bronchus and lung	UKBB	2018	361194	European
	FinnGen Biobank	2020	96499	European
	ILCCO	2014	27209	European
Breast	UKBB	2018	462933	European
	FinnGen Biobank	2020	96499	European
	BCAC	2017	228951	European
Pancreas	PanScan1	2009	3,835	European
	FinnGen Biobank	2020	96499	European
Colon	UKBB	2018	462933	European
	FinnGen Biobank	2020	96499	European
Rectum	UKBB	2018	463010	European
	FinnGen Biobank	2020	96499	European
Kidney	UKBB	2018	463010	European
	FinnGen Biobank	2020	96499	European
Bladder	UKBB	2018	462933	European
	FinnGen Biobank	2020	96499	European
Prostate	UKBB	2018	463010	European
	FinnGen Biobank	2020	96499	European
	PRACTICAL	2018	140254	European
Ovary	UKBB	2018	463010	European
	FinnGen Biobank	2020	96499	European
	OCAC	2017	66450	European
Uterus/endometrium	UKBB	2018	462933	European
	FinnGen Biobank	2020	96499	European

Abbreviations: UKBB, United Kingdom, biobank; ILCCO, international lung cancer consortium; BCAC, breast cancer association consortium; PanScan1, Pancreatic Cancer Cohort Consortium GWAS; PRACTICAL, prostate cancer association group to investigate cancer-associated alterations in the genome; OCAC, ovarian cancer association consortium.

### Statistical Analysis

An MR analysis was performed utilizing 10 vitamin C–related SNPs as IVs to evaluate the association of vitamin C with overall cancer risk and site-specific cancer risk. We used the inverse-variance weight (IVW) method with random effects to implement the primary MR analysis. The odds ratio (OR) and 95% CI for risk of overall cancer and site-specific cancer were estimated.

We then performed sensitivity analyses, including MR-Egger regression, simple mode, weighted median, and weighted mode methods to determine whether the IVs can influence cancer only through their effect on vitamin C. To test bias from pleiotropic effects, we used MR-Egger regression. In addition, the slope coefficient from an Egger regression provided a reliable estimate of any causal effect (Bowden et al., 2015). The weighted median method could provide a consistent assessment of the finding if more than half of the weight comes from valid IVs (Bowden et al., 2016). When the most common horizontal pleiotropy value was zero regardless of the type of horizontal pleiotropy, we performed the simple mode method to offer a consistent assessment (Bowden et al., 2016). In addition, the weighted mode requires that the largest subset of instruments identifying the same causal effect estimates is contributed by valid IVs (Hartwig et al., 2017). A pleiotropy test was also performed to test whether IVs had horizontal pleiotropy. We also applied the MR-Pleiotropy Residual Sum and Outlier (MR-PRESSO) analysis to determine the horizontal pleiotropy and correct the potential outliers (Verbanck et al., 2018). In addition, we utilized Cochran’s Q test on the IVW and MR-Egger estimates to test the heterogeneity of the causal estimates. We also used a leave-one-out sensitivity test to test whether the MR outcome was sensitive to its related IV. MR and sensitivity analyses were performed in R (version 4.0.2) using the Two-Sample MR package (version 0.5.5) and the MRPRESSO package (version 1.0).

## Results

Our findings did not support the causal association between vitamin C and the risk of overall cancer in the UK Biobank and FinnGen Biobank (OR: 0.998, 95% CI: 0.992–1.004, *p* = 0.452, and OR: 1.046, 95% CI: 0.839–1.304, *p* = 0.692, respectively). The results of MR-Egger, weighted median, simple mode, and weighted mode analyses were similar to those of the IVW (Table 3). In sensitivity analysis, heterogeneity was not detected (Supplementary Table S1). In addition, we did not detect horizontal pleiotropy *via* pleiotropy tests and MR-PRESSO analysis (Supplementary Tables S2, S3). A scatter plot of the association between vitamin C and overall cancer is shown in Supplementary Figure S1.

**TABLE 3:** Associations between genetically predicted vitamin C and risk of cancer.

Type of cancer	Data source	Number of SNPs	Inverse variance weighted		MR-Egger		Simple mode		Weighted median		Weighted mode	
			Or (95%CI)	P	Or (95%CI)	P	Or (95%CI)	P	Or (95%CI)	P	Or (95%CI)	P
Overall cancer	UKBB	10	0.998 (0.992–1.004)	0.452	1.003 (0.994–1.012)	0.562	0.991 (0.977–1.006)	0.28	1.002 (0.994–1.009)	0.663	1.003 (0.995–1.011)	0.492
	FinnGen Biobank	7	1.046 (0.839–1.304)	0.692	1.107 (0.779–1.573)	0.596	1.073 (0.731–1.574)	0.732	1.076 (0.848–1.367)	0.546	1.077 (0.834–1.392)	0.59
Bronchus and lung	UKBB	10	0.999 (0.998–1.001)	0.323	1.000 (0.997–1.003)	0.982	0.996 (0.993–1.000)	0.058	1.000 (0.998–1.002)	0.795	1.000 (0.998–1.002)	0.889
	FinnGen Biobank	7	1.035 (0.355–3.017)	0.95	2.274 (0.522–9.903)	0.323	1.414 (0.388–5.156)	0.618	1.517 (0.582–3.957)	0.394	1.590 (0.561–4.505)	0.416
	ILCCO	9	1.014 (0.690–1.491)	0.943	1.259 (0.678–2.340)	0.49	1.174 (0.757–1.820)	0.494	1.075 (0.830–1.391)	0.586	1.078 (0.829–1.402)	0.592
Breast	UKBB	10	1.002 (0.999–1.005)	0.15	1.002 (0.998–1.007)	0.362	0.999 (0.993–1.006)	0.826	1.002 (0.998–1.006)	0.292	1.002 (0.998–1.007)	0.301
	FinnGen Biobank	7	0.842 (0.470–1.507)	0.562	0.516 (0.246–1.079)	0.139	0.780 (0.381–1.598)	0.523	0.669 (0.430–1.041)	0.075	0.652 (0.420–1.012)	0.105
	BCAC	8	1.046 (0.931–1.176)	0.447	1.039 (0.862–1.252)	0.704	1.002 (0.826–1.215)	0.985	1.042 (0.948–1.146)	0.389	1.053 (0.951–1.166)	0.355
Pancreas	PanScan1	4	1.440 (0.556–3.731)	0.452	0.612 (0.058–6.485)	0.723	1.253 (0.289–5.439)	0.783	1.249 (0.417–3.746)	0.691	1.173 (0.339–4.060)	0.818
	FinnGen Biobank	7	0.783 (0.230–2.672)	0.697	0.873 (0.120–6.358)	0.898	1.043 (0.130–8.376)	0.969	0.721 (0.177–2.925)	0.647	0.897 (0.210–3.830)	0.888
Colon	UKBB	6	0.997 (0.994–0.999)	0.003	1.000 (0.987–1.013)	0.986	0.997 (0.993–1.001)	0.167	0.997 (0.994–1.000)	0.048	0.997 (0.993–1.001)	0.164
	FinnGen Biobank	7	0.624 (0.269–1.445)	0.271	0.590 (0.151–2.297)	0.481	0.633 (0.138–2.889)	0.576	0.616 (0.252–1.503)	0.287	0.619 (0.256–1.497)	0.328
Rectum	UKBB	6	0.998 (0.996–1.001)	0.164	0.993 (0.980–1.006)	0.342	0.998 (0.993–1.002)	0.39	0.998 (0.995–1.001)	0.157	0.997 (0.993–1.001)	0.227
	FinnGen Biobank	7	0.831 (0.278–2.490)	0.741	1.287 (0.252–6.556)	0.774	0.361 (0.064–2.027)	0.291	0.971 (0.273–3.457)	0.964	1.055 (0.252–4.418)	0.944
Kidney	UKBB	5	1.001 (0.999–1.003)	0.348	1.008 (0.996–1.019)	0.296	1.002 (0.998–1.005)	0.405	1.002 (0.999–1.004)	0.168	1.002 (0.999–1.006)	0.319
	FinnGen Biobank	7	1.019 (0.258–4.032)	0.979	3.268 (0.566–18.852)	0.243	1.411 (0.226–8.812)	0.725	1.875 (0.555–6.342)	0.312	1.936 (0.606–6.192)	0.308
Bladder	UKBB	5	0.999 (0.997–1.002)	0.568	1.005 (0.993–1.017)	0.475	1.000 (0.996–1.004)	0.921	1.000 (0.998–1.003)	0.869	1.001 (0.997–1.004)	0.759
	FinnGen Biobank	7	1.177 (0.316–4.384)	0.808	3.023 (0.489–18.67)	0.287	1.916 (0.276–13.28)	0.535	1.694 (0.527–5.442)	0.376	2.039 (0.638–6.515)	0.275
Prostate	UKBB	9	1.000 (0.996–1.004)	0.966	1.000 (0.989–1.011)	0.995	1.002 (0.995–1.009)	0.59	1.000 (0.995–1.005)	0.937	1.001 (0.996–1.007)	0.661
	FinnGen Biobank	7	1.393 (0.899–2.156)	0.138	1.491 (0.779–2.854)	0.282	1.389 (0.612–3.150)	0.462	1.396 (0.826–2.362)	0.213	1.428 (0.831–2.455)	0.245
	PRACTICAL	10	0.966 (0.886–1.054)	0.438	0.974 (0.850–1.116)	0.715	0.984 (0.823–1.177)	0.863	0.980 (0.880–1.091)	0.709	0.986 (0.876–1.108)	0.814
Ovary	UKBB	5	0.998 (0.996–1.000)	0.04	0.996 (0.984–1.007)	0.526	0.997 (0.994–1.001)	0.192	0.998 (0.995–1.000)	0.052	0.997 (0.994–1.000)	0.17
	FinnGen Biobank	7	0.957 (0.260–3.519)	0.947	0.470 (0.068–3.254)	0.479	1.247 (0.124–12.539)	0.857	0.685 (0.148–3.172)	0.628	0.655 (0.117–3.652)	0.646
	OCAC	8	0.928 (0.792–1.088)	0.358	0.801 (0.638–1.006)	0.105	1.093 (0.797–1.498)	0.599	0.891 (0.739–1.074)	0.227	0.857 (0.712–1.033)	0.149
Uterus/endometrium	UKBB	5	1.000 (0.998–1.002)	0.809	1.004 (0.992–1.016)	0.527	1.000 (0.996–1.003)	0.973	1.000 (0.997–1.003)	0.948	1.000 (0.997–1.003)	0.889
	FinnGen Biobank	7	1.230 (0.488–3.101)	0.661	2.922 (0.743–11.488)	0.185	0.862 (0.134–5.553)	0.881	1.864 (0.607–5.723)	0.276	1.940 (0.661–5.696)	0.273

Abbreviations: SNP, single-nucleotide polymorphism; OR, odds ratio; CI, confidence interval; UKBB, UK biobank; ILCCO, international lung cancer consortium; BCAC, breast cancer association consortium; PanScan1, Pancreatic Cancer Cohort Consortium GWAS; PRACTICAL, prostate cancer association group to investigate cancer-associated alterations in the genome; OCAC, ovarian cancer association consortium.

**FIGURE 2:**
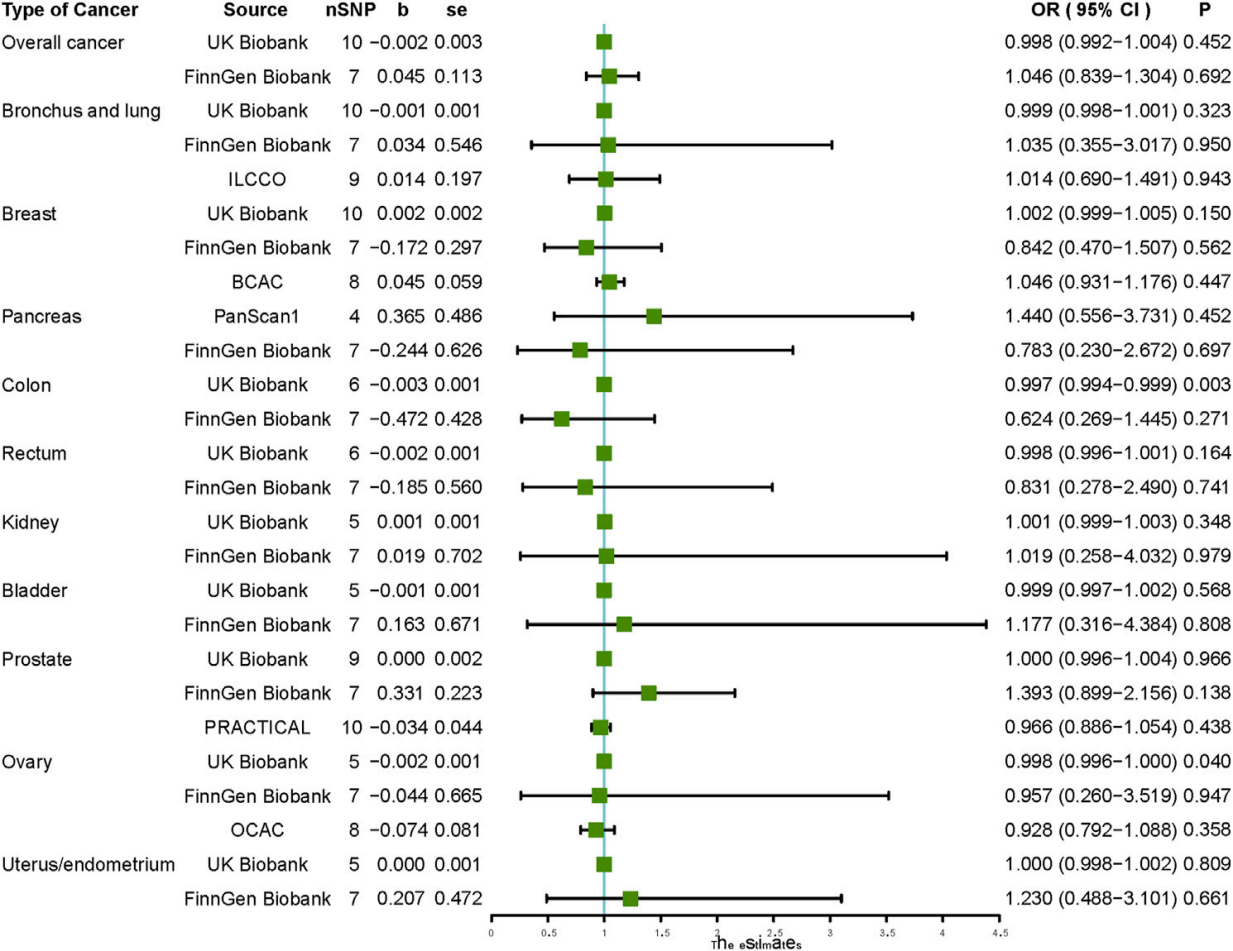
Causal effect estimates of vitamin C on cancer outcomes. SNP, single-nucleotide polymorphism; OR, odds ratio; CI, confidence interval; UKBB, UKn Biobank; ILCCO, International Lung Cancer Consortium; BCAC, Breast Cancer Association Consortium; PanScan1, Pancreatic Cancer Cohort Consortium GWAS; PRACTICAL, Prostate Cancer Association group To Investigate Cancer-Associated Alterations in the Genome; OCAC, Ovarian Cancer Association Consortium.

When analyzing the causal relationship between vitamin C and different types of cancer, our IVW results did not support the causal association between vitamin C and the risk of any of the ten types of cancer, including malignant neoplasm of the bronchus and lung, breast, pancreas, colon, rectum, kidney, bladder, prostate, ovary, and endometrium (Figure 2). Using MR-Egger, weighted median, simple mode, and weighted mode methods, we obtained similar results to those of IVW, which did not support the causal association between vitamin C and any type of cancer (Table 3).

In sensitivity analysis of vitamin C and site-specific cancer, our results did not reveal substantial heterogeneity except for that in lung cancer and breast cancer (Supplementary Table S1), and a pleiotropy test using the MR-Egger intercept did not detect any pleiotropy across the studies (Supplementary Table S2). In MR-PRESSO analysis, we did not detect horizontal pleiotropy except for the association between vitamin C and lung cancer in the ILCCO dataset (Supplementary Table S3). We further found that rs174547 was a potential outlier (*p* < 0.01), and after omitting rs174547, vitamin C was still not associated with the risk of lung cancer (OR: 0.999, 95% CI: 0.998–1.001, *p* = 0.481). Details of the leave-one-out sensitivity test are displayed in Supplementary Table S4. A scatter plot of the association between vitamin C and 10 types of site-specific cancer is shown in Supplementary Figures S2–S11.

## Discussion

The prevention and therapeutic effects of vitamin C on cancer have been debated for decades. In this MR study, we demonstrated that vitamin C was not causally associated with the risk of cancer. In particular, our findings did not support the causal association between vitamin C and the risk of overall cancer or any specific type of cancer, including colon cancer and ovarian cancer, and the risk of malignant neoplasm of the bronchus and lung, breast, pancreas, colon, rectum, kidney, bladder, prostate, ovary, and uterine/endometrium. MR-Egger regression, simple mode, weighted median, and weighted mode methods showed similar findings. In addition, in sensitivity analysis, heterogeneity and horizontal pleiotropy were not detected in most of our studies.

In general, our findings were in line with those of previous studies aimed at investigating the association between vitamin C and cancer. A recent systematic review included 19 clinical trials that did not support the protective effect of vitamin C supplementation in patients with cancer on their clinical status and overall survival (van Gorkom et al., 2019). One meta-analysis included three studies that indicated vitamin C had no significant effect on lung cancer incidence (Cortés-Jofré et al., 2020). A meta-analysis that included 20 observational studies did not support the relationship between vitamin C intake and the risk of pancreatic cancer (Hua et al., 2016). Another meta-analysis of three prospective cohort studies did not observe an association between vitamin C intake and the risk of renal cell carcinoma (Jia et al., 2015b). A meta-analysis involving 16 studies indicated no effect of vitamin C on reducing the risk of ovarian cancer (RR: 0.95, 95% CI: 0.81–1.11) (Long et al., 2020). In addition, for prostate cancer, a meta-analysis that summarized nine RCTs found no relationship between vitamin C intake and the incidence of prostate cancer (RR: 1.45, 95% CI: 0.92–2.29) (Jiang et al., 2010). However, some of our results were inconsistent with those of several observational studies. At the same time, a meta-analysis involving 13 cohort studies suggested that supplementary intake of vitamin C could reduce the risk of colon cancer (RR: 0.81, 95% CI: 0.71–0.92) (Park et al., 2010). Moreover, targeting female-specific tumors, supplementary intake of vitamin C could reduce the risk of cervical neoplasia (OR: 0.58, 95% CI: 0.44–0.75) (Cao et al., 2016). In addition, another meta-analysis included 12 studies suggesting that vitamin C could prevent endometrial cancer (OR: 0.85, 95% CI: 0.73–0.98) (Bandera et al., 2009). But, most of the available clinical studies were cross-sectional, case-control, and cohort studies, the results of which were easily affected by known and unknown confounding factors and reverse causality (Bandera et al., 2009; Bo et al., 2016). Heterogeneity was detected in most of the studies. In addition, case-control studies were also affected by recall and selection biases. The current study used MR analysis, which utilized genetically predicted SNPs as IVs for the exposure, to explore the causal relationship between exposure and outcome that could minimize the effect of the potential confounders and reverse causality. Therefore, the findings of high-quality MR studies could be more convincing than those of the aforementioned observational studies. One previous MR study assessed the relationships between plasma vitamin C levels and five types of cancer, including lung, breast, prostate, colon, and rectal cancer. Similar to our findings, the use of vitamin C supplements was not causally associated with the risk of these types of cancer (Fu et al., 2021).

Previous experiments have well-investigated the therapeutic effects of vitamin C and confirmed that vitamin C is capable of killing cancer cells *in vitro* and shrinking tumor size *in vivo*. Multiple pathways might be involved in the antitumor effect of vitamin C, including targeting redox imbalance, acting as an epigenetic regulator and modifying hypoxia-inducible factor 1 (HIF1) signaling (Cimmino et al., 2017; Ngo et al., 2019). But, there were few experimental studies that supported the prevention effect of vitamin C on the risk of cancer (Reczek and Chandel, 2015). In that case, vitamin C seemed to be unable to reduce cancer incidence but could act as an additional therapeutic agent for cancer treatment. Moreover, even with the usage of supplementary vitamin C, the plasma vitamin C concentration among a healthy population was likely unable to reach the dose of vitamin C utilized in experiments *in vivo* and *in vitro*, which led to the fact that supplementary vitamin C intake failed to reduce the risk of cancer in the general population.

The current study had several advantages and disadvantages. A major strength of this study was the MR study design, which could diminish confounding and reverse causality. Second, in this study, we broadly assessed the causal relationship of plasma vitamin C concentrations with the overall and a wide range of different types of cancer with a large number of cancer cases. Third, for each type of cancer, we validated our results in at least two datasets, which improved the robustness of our findings. However, there were also several limitations to the present study. First, the sample sizes of several types of cancer cases were small, resulting in low precision in the assessment. In that case, we might have ignored some weak associations. To deal with the problem, for those MR results generated from GWASs with small sample sizes, we validated the findings using another GWAS with a larger sample size. It should also be noted that the analyses are limited by the potential of the GWAS studies from which the IVs have been identified. In addition, in our study, the IVs were extracted from the largest GWAS study of vitamin C, and the F-statistics for the IVs were over 10, which could reduce the potential weak instrument bias. Second, our analyses were based on GWAS of European ancestry, and the results may be different in different ancestries; hence, our results might not be generalizable to all populations. Third, our study could only determine the causal relationship between circulating vitamin C levels and cancer risk but did not investigate the therapeutic effect of vitamin C on cancer.

## Conclusion

This MR study did not support the causal association between vitamin C and the risk of overall or any specific types of cancer. Although previous observational studies and experiments confirmed an anticancer effect of vitamin C, these results might be influenced by confounding factors and were unable to illustrate the actual connection between vitamin C and cancer. Therefore, further studies are warranted to explore the relationship between vitamin C and the risk of cancer.

## Data Availability

Publicly available datasets were analyzed in this study. These data can be found here: Summary statistics of GWAS on overall cancer and nine site-specific cancers, including lung, breast, colon, rectum, kidney, bladder, prostate, ovarian, and uterine/endometrial cancer, were obtained from the United Kingdom Biobank dataset upon application (https://www.ukbiobank.ac.uk/). GWAS summary-level data on overall cancer and malignant neoplasm of the bronchus and lung, breast, pancreas, colon, rectum, kidney, bladder, prostate, ovary, and corpus uteri from the FinnGen consortium are available at https://finngen.gitbook.io/documentation/. GWAS summary-level data on lung cancer, breast cancer, pancreatic cancer, prostate cancer, and ovarian cancer were obtained from ILCCO, BCAC, PanScan1, PRACTICAL, and OCAC *via* https://gwas.mrcieu.ac.uk/datasets/, respectively.

## Supplementary Material

Supplementary Figure S1 Scatter plot of vitamin C on overall cancer. **(A)** Scatter plot of vitamin C on overall cancer in UK Biobank. **(B)** Scatter plot of vitamin C on overall cancer in FinnGen Biobank.

Supplementary Figure S2 Scatter plot of vitamin C on bronchus and lung cancer. **(A)** Scatter plot of vitamin C on bronchus and lung cancer in UK Biobank. **(B)** Scatter plot of vitamin C on bronchus and lung cancer in FinnGen Biobank. **(C)** Scatter plot of vitamin C on lung cancer in ILCCO. ILCCO, International Lung Cancer Consortium.

Supplementary Figure S3 Scatter plot of vitamin C on breast cancer. **(A)** Scatter plot of vitamin C on breast cancer in UK Biobank. **(B)** Scatter plot of vitamin C on breast cancer in FinnGen Biobank. **(C)** Scatter plot of vitamin C on breast cancer in BCAC. BCAC, Breast Cancer Association Consortium.

Supplementary Figure S4 Scatter plot of vitamin C on pancreatic cancer. **(A)** Scatter plot of vitamin C on pancreatic cancer in PanScan1. **(B)** Scatter plot of vitamin C on pancreatic cancer in FinnGen Biobank. PanScan1, Pancreatic Cancer Cohort Consortium GWAS.

Supplementary Figure S5 Scatter plot of vitamin C on colon cancer. **(A)** Scatter plot of vitamin C on colon cancer in UK Biobank. **(B)** Scatter plot of vitamin C on colon cancer in FinnGen Biobank.

Supplementary Figure S6 Scatter plot of vitamin C on rectal cancer. **(A)** Scatter plot of vitamin C on rectal cancer in UK Biobank. **(B)** Scatter plot of vitamin C on rectal cancer in FinnGen Biobank.

Supplementary Figure 7 Scatter plot of vitamin C on kidney cancer. **(A)** Scatter plot of vitamin C on kidney cancer in UK Biobank. **(B)** Scatter plot of vitamin C on kidney cancer in FinnGen Biobank.

Supplementary Figure S8 Scatter plot of vitamin C on bladder cancer. **(A)** Scatter plot of vitamin C on bladder cancer in UK Biobank. **(B)** Scatter plot of vitamin C on bladder cancer in FinnGen Biobank.

Supplementary Figure S9 Scatter plot of vitamin C on prostate cancer. **(A)** Scatter plot of vitamin C on prostate cancer in UK Biobank. **(B)** Scatter plot of vitamin C on prostate cancer in FinnGen Biobank. **(C)** Scatter plot of vitamin C on prostate cancer in PRACTICAL. PRACTICAL, Prostate Cancer Association group To Investigate Cancer-Associated Alterations in the Genome.

Supplementary Figure S10 Scatter plot of vitamin C on ovarian cancer. **(A)** Scatter plot of vitamin C on ovarian cancer in UK Biobank. **(B)** Scatter plot of vitamin C on ovarian cancer in FinnGen Biobank. **(C)** Scatter plot of vitamin C on ovarian cancer in OCAC. OCAC, Ovarian Cancer Association Consortium.

Supplementary Figure S11 Scatter plot of vitamin C on endometrial cancer. **(A)** Scatter plot of vitamin C on endometrial cancer in UK Biobank. **(B)** Scatter plot of vitamin C on endometrial cancer in FinnGen Biobank.
