# Supplementary material for: The Association Between Vitamin C and Cancer: A Two-Sample Mendelian Randomization Study
Source: Front Genet. 2022 May 5;13:868408. doi: 10.3389/fgene.2022.868408 (PMC9117647; doi:10.3389/fgene.2022.868408)
Supplement: Supplementary file 1 [file DataSheet1.ZIP › Supplementary Table S1.docx]

**Supplementary Table S1.** Heterogeneity test of IVW and MR-Egger estimates of the associations between vitamin C and risk of cancer.

| **Outcome** | **Source** | **Exposure** | **Method** | **Q** | **Q_df** | **Q_pval** |
| --- | --- | --- | --- | --- | --- | --- |
| Overall cancer | UK Biobank | Vitamin C | Inverse variance weighted | 5.610782 | 9 | 0.778152 |
| Overall cancer | UK Biobank | Vitamin C | MR Egger | 3.624141 | 8 | 0.889344 |
| Overall cancer | FinnGen Biobank | Vitamin C | Inverse variance weighted | 7.321937 | 6 | 0.292098 |
| Overall cancer | FinnGen Biobank | Vitamin C | MR Egger | 7.060278 | 5 | 0.216196 |
| Bronchus and lung | UK Biobank | Vitamin C | Inverse variance weighted | 7.467998 | 9 | 0.588514 |
| Bronchus and lung | UK Biobank | Vitamin C | MR Egger | 6.839629 | 8 | 0.55403 |
| Bronchus and lung | FinnGen Biobank | Vitamin C | Inverse variance weighted | 11.08722 | 6 | 0.085718 |
| Bronchus and lung | FinnGen Biobank | Vitamin C | MR Egger | 7.90423 | 5 | 0.161593 |
| Lung | ILCCO | Vitamin C | Inverse variance weighted | 29.73218 | 8 | 0.000236 |
| Lung | ILCCO | Vitamin C | MR Egger | 26.75669 | 7 | 0.000369 |
| Breast | UK Biobank | Vitamin C | Inverse variance weighted | 6.285189 | 9 | 0.711071 |
| Breast | UK Biobank | Vitamin C | MR Egger | 6.281345 | 8 | 0.615749 |
| Breast | FinnGen Biobank | Vitamin C | Inverse variance weighted | 15.12294 | 6 | 0.019322 |
| Breast | FinnGen Biobank | Vitamin C | MR Egger | 9.30599 | 5 | 0.097464 |
| Breast | BCAC | Vitamin C | Inverse variance weighted | 18.1092 | 7 | 0.011487 |
| Breast | BCAC | Vitamin C | MR Egger | 18.07739 | 6 | 0.006042 |
| Pancreas | PanScan1 | Vitamin C | Inverse variance weighted | 0.979185 | 3 | 0.806288 |
| Pancreas | PanScan1 | Vitamin C | MR Egger | 0.375457 | 2 | 0.82884 |
| Pancreas | FinnGen Biobank | Vitamin C | Inverse variance weighted | 6.665181 | 6 | 0.352923 |
| Pancreas | FinnGen Biobank | Vitamin C | MR Egger | 6.637341 | 5 | 0.249039 |
| Colon | UK Biobank | Vitamin C | Inverse variance weighted | 2.764511 | 5 | 0.736235 |
| Colon | UK Biobank | Vitamin C | MR Egger | 2.521272 | 4 | 0.640831 |
| Colon | FinnGen Biobank | Vitamin C | Inverse variance weighted | 7.932721 | 6 | 0.243074 |
| Colon | FinnGen Biobank | Vitamin C | MR Egger | 7.913571 | 5 | 0.161064 |
| Rectum | UK Biobank | Vitamin C | Inverse variance weighted | 3.058676 | 5 | 0.69094 |
| Rectum | UK Biobank | Vitamin C | MR Egger | 2.334136 | 4 | 0.674561 |
| Rectum | FinnGen Biobank | Vitamin C | Inverse variance weighted | 0.774137 | 6 | 0.992749 |
| Rectum | FinnGen Biobank | Vitamin C | MR Egger | 0.268649 | 5 | 0.998191 |
| Kidney | UK Biobank | Vitamin C | Inverse variance weighted | 2.126556 | 4 | 0.712495 |
| Kidney | UK Biobank | Vitamin C | MR Egger | 0.897677 | 3 | 0.825988 |
| Kidney | FinnGen Biobank | Vitamin C | MR Egger | 6.711129 | 5 | 0.243026 |
| Kidney | FinnGen Biobank | Vitamin C | Inverse variance weighted | 10.89552 | 6 | 0.09166 |
| Bladder | UK Biobank | Vitamin C | Inverse variance weighted | 3.23698 | 4 | 0.518979 |
| Bladder | UK Biobank | Vitamin C | MR Egger | 2.3635 | 3 | 0.500465 |
| Bladder | FinnGen Biobank | Vitamin C | Inverse variance weighted | 11.98296 | 6 | 0.06235 |
| Bladder | FinnGen Biobank | Vitamin C | MR Egger | 8.695769 | 5 | 0.121832 |
| Prostate | UK Biobank | Vitamin C | Inverse variance weighted | 3.87341 | 8 | 0.86836 |
| Prostate | UK Biobank | Vitamin C | MR Egger | 3.873344 | 7 | 0.794245 |
| Prostate | FinnGen Biobank | Vitamin C | Inverse variance weighted | 3.711654 | 6 | 0.71563 |
| Prostate | FinnGen Biobank | Vitamin C | MR Egger | 3.633546 | 5 | 0.603284 |
| Prostate | PRACTICAL | Vitamin C | Inverse variance weighted | 4.791737 | 9 | 0.852072 |
| Prostate | PRACTICAL | Vitamin C | MR Egger | 4.768973 | 8 | 0.781959 |
| Ovary | UK Biobank | Vitamin C | Inverse variance weighted | 0.292211 | 4 | 0.990311 |
| Ovary | UK Biobank | Vitamin C | MR Egger | 0.172009 | 3 | 0.981976 |
| Ovary | FinnGen Biobank | Vitamin C | Inverse variance weighted | 3.968096 | 6 | 0.680994 |
| Ovary | FinnGen Biobank | Vitamin C | MR Egger | 3.020598 | 5 | 0.69681 |
| Ovary | OCAC | Vitamin C | Inverse variance weighted | 7.481629 | 7 | 0.38051 |
| Ovary | OCAC | Vitamin C | MR Egger | 4.542398 | 6 | 0.60369 |
| Uterus/endometrium | UK Biobank | Vitamin C | Inverse variance weighted | 0.930103 | 4 | 0.920203 |
| Uterus/endometrium | UK Biobank | Vitamin C | MR Egger | 0.33682 | 3 | 0.952961 |
| Corpus uteri | FinnGen Biobank | Vitamin C | Inverse variance weighted | 4.0868 | 6 | 0.664931 |
| Corpus uteri | FinnGen Biobank | Vitamin C | MR Egger | 1.260427 | 5 | 0.938951 |

Abbreviations: ILCCO, International Lung Cancer Consortium; BCAC, Breast Cancer Association Consortium; PanScan1, Pancreatic Cancer Cohort Consortium GWAS; PRACTICAL, Prostate Cancer Association group To Investigate Cancer Associated Alterations in the Genome; OCAC, Ovarian Cancer Association Consortium.
