# Supplementary material for: The Association Between Vitamin C and Cancer: A Two-Sample Mendelian Randomization Study
Source: Front Genet. 2022 May 5;13:868408. doi: 10.3389/fgene.2022.868408 (PMC9117647; doi:10.3389/fgene.2022.868408)
Supplement: Supplementary file 1 [file DataSheet1.ZIP › Supplementary Table S2.docx]

**Supplementary Table S2.** Pleiotropy test of the associations between vitamin C and risk of cancer.

| **Outcome** | **Source** | **Exposure** | **Egger_intercept** | **se** | **pval** |
| --- | --- | --- | --- | --- | --- |
| Overall cancer | UK Biobank | Vitamin C | -0.00047 | 0.000334 | 0.19636 |
| Overall cancer | FinnGen Biobank | Vitamin C | -0.00536 | 0.012455 | 0.684778 |
| Bronchus and lung | UK Biobank | Vitamin C | -7.51E-05 | 9.48E-05 | 0.45082 |
| Bronchus and lung | FinnGen Biobank | Vitamin C | -0.07365 | 0.051903 | 0.215125 |
| Lung | ILCCO | Vitamin C | -0.01956 | 0.022173 | 0.406895 |
| Breast | UK Biobank | Vitamin C | -1.13E-05 | 0.000181 | 0.952084 |
| Breast | FinnGen Biobank | Vitamin C | 0.046447 | 0.026273 | 0.137323 |
| Breast | BCAC | Vitamin C | 0.000715 | 0.006961 | 0.921496 |
| Pancreas | PanScan1 | Vitamin C | 0.055079 | 0.070887 | 0.518471 |
| Pancreas | FinnGen Biobank | Vitamin C | -0.0102 | 0.070452 | 0.890512 |
| Colon | UK Biobank | Vitamin C | -0.00015 | 0.000295 | 0.647726 |
| Colon | FinnGen Biobank | Vitamin C | 0.005319 | 0.048357 | 0.916691 |
| Rectum | UK Biobank | Vitamin C | 0.000249 | 0.000292 | 0.442616 |
| Rectum | FinnGen Biobank | Vitamin C | -0.04087 | 0.057483 | 0.50888 |
| Kidney | UK Biobank | Vitamin C | -0.00029 | 0.00026 | 0.348507 |
| Kidney | FinnGen Biobank | Vitamin C | -0.10953 | 0.062035 | 0.137718 |
| Bladder | UK Biobank | Vitamin C | -0.00024 | 0.000258 | 0.418938 |
| Bladder | FinnGen Biobank | Vitamin C | -0.08882 | 0.064605 | 0.227602 |
| Prostate | UK Biobank | Vitamin C | 2.42E-06 | 0.000298 | 0.99374 |
| Prostate | FinnGen Biobank | Vitamin C | -0.00641 | 0.02293 | 0.79107 |
| Prostate | PRACTICAL | Vitamin C | -0.00075 | 0.004994 | 0.883806 |
| Ovary | UK Biobank | Vitamin C | 8.88E-05 | 0.000256 | 0.751709 |
| Ovary | FinnGen Biobank | Vitamin C | 0.066411 | 0.068226 | 0.375063 |
| Ovary | OCAC | Vitamin C | 0.014495 | 0.008455 | 0.137279 |
| Uterus/endometrium | UK Biobank | Vitamin C | -0.0002 | 0.000264 | 0.497249 |
| Corpus uteri | FinnGen Biobank | Vitamin C | -0.08161 | 0.048543 | 0.153557 |

Abbreviations: ILCCO, International Lung Cancer Consortium; BCAC, Breast Cancer Association Consortium; PanScan1, Pancreatic Cancer Cohort Consortium GWAS; PRACTICAL, Prostate Cancer Association group To Investigate Cancer Associated Alterations in the Genome; OCAC, Ovarian Cancer Association Consortium.
