# Supplementary material for: The Association Between Vitamin C and Cancer: A Two-Sample Mendelian Randomization Study
Source: Front Genet. 2022 May 5;13:868408. doi: 10.3389/fgene.2022.868408 (PMC9117647; doi:10.3389/fgene.2022.868408)
Supplement: Supplementary file 1 [file DataSheet1.ZIP › Supplementary Table S3.docx]

**Supplementary Table S3.** MR-PRESSO sensitivity test of the associations between vitamin C and risk of cancer.

| **Type of cancer** | **Source** | **MR-PRESSO p value** |
| --- | --- | --- |
| Overall cancer | UK Biobank | 0.576 |
| Overall cancer | FinnGen Biobank | 0.423 |
| Bronchus and lung | UK Biobank | 0.65 |
| Bronchus and lung | FinnGen Biobank | 0.174 |
| Lung | ILCCO | 0.002 |
| Breast | UK Biobank | 0.776 |
| Breast | FinnGen Biobank | 0.075 |
| Breast | BCAC | 0.05 |
| Pancreas | PanScan1 | 0.794 |
| Pancreas | FinnGen Biobank | 0.468 |
| Colon | UK Biobank | 0.792 |
| Colon | FinnGen Biobank | 0.368 |
| Rectum | UK Biobank | 0.699 |
| Rectum | FinnGen Biobank | 0.987 |
| Kidney | UK Biobank | 0.7 |
| Kidney | FinnGen Biobank | 0.167 |
| Bladder | UK Biobank | 0.522 |
| Bladder | FinnGen Biobank | 0.158 |
| Prostate | UK Biobank | 0.879 |
| Prostate | FinnGen Biobank | 0.776 |
| Prostate | PRACTICAL | 0.898 |
| Ovary | UK Biobank | 0.988 |
| Ovary | FinnGen Biobank | 0.733 |
| Ovary | OCAC | 0.424 |
| Uterine/endometrium | UK Biobank | 0.91 |
| Corpus uteri | FinnGen Biobank | 0.649 |

Abbreviations: ILCCO, International Lung Cancer Consortium; BCAC, Breast Cancer Association Consortium; PanScan1, Pancreatic Cancer Cohort Consortium GWAS; PRACTICAL, Prostate Cancer Association group To Investigate Cancer Associated Alterations in the Genome; OCAC, Ovarian Cancer Association Consortium.
