# Supplementary material for: The Association Between Vitamin C and Cancer: A Two-Sample Mendelian Randomization Study
Source: Front Genet. 2022 May 5;13:868408. doi: 10.3389/fgene.2022.868408 (PMC9117647; doi:10.3389/fgene.2022.868408)
Supplement: Supplementary file 1 [file DataSheet1.ZIP › Supplementary Table S4.docx]

**Supplementary Table S4.** Leave-one-out sensitivity test of the associations between vitamin C and risk of cancer.

| **Outcome** | **Source** | **Exposure** | **SNP** | **b** | **se** | **p** |
| --- | --- | --- | --- | --- | --- | --- |
| Overall cancer | UK Biobank | Vitamin C | rs10051765 | -0.00205 | 0.00299 | 0.492886 |
| Overall cancer | UK Biobank | Vitamin C | rs10136000 | -0.00211 | 0.002987 | 0.480764 |
| Overall cancer | UK Biobank | Vitamin C | rs117885456 | -0.0017 | 0.003014 | 0.571793 |
| Overall cancer | UK Biobank | Vitamin C | rs13028225 | -0.00071 | 0.003158 | 0.82174 |
| Overall cancer | UK Biobank | Vitamin C | rs174547 | -0.00173 | 0.002982 | 0.562191 |
| Overall cancer | UK Biobank | Vitamin C | rs2559850 | -0.00195 | 0.003071 | 0.524925 |
| Overall cancer | UK Biobank | Vitamin C | rs33972313 | -0.00739 | 0.004047 | 0.067704 |
| Overall cancer | UK Biobank | Vitamin C | rs56738967 | -0.0017 | 0.002994 | 0.57024 |
| Overall cancer | UK Biobank | Vitamin C | rs6693447 | -0.00246 | 0.002997 | 0.411501 |
| Overall cancer | UK Biobank | Vitamin C | rs9895661 | -0.00244 | 0.003029 | 0.42112 |
| Overall cancer | UK Biobank | Vitamin C | All | -0.00221 | 0.002934 | 0.452166 |
| Overall cancer | FinnGen Biobank | Vitamin C | rs10051765 | 0.080096 | 0.10849 | 0.46034 |
| Overall cancer | FinnGen Biobank | Vitamin C | rs10136000 | 0.050788 | 0.126007 | 0.686905 |
| Overall cancer | FinnGen Biobank | Vitamin C | rs13028225 | 0.034007 | 0.138618 | 0.8062 |
| Overall cancer | FinnGen Biobank | Vitamin C | rs174547 | 0.07875 | 0.10637 | 0.459095 |
| Overall cancer | FinnGen Biobank | Vitamin C | rs33972313 | 0.017534 | 0.175142 | 0.920254 |
| Overall cancer | FinnGen Biobank | Vitamin C | rs56738967 | 0.03875 | 0.126537 | 0.759428 |
| Overall cancer | FinnGen Biobank | Vitamin C | rs6693447 | -0.00333 | 0.105085 | 0.974724 |
| Overall cancer | FinnGen Biobank | Vitamin C | All | 0.044528 | 0.11254 | 0.692352 |
| Bronchus and lung | UK Biobank | Vitamin C | rs10051765 | -0.00092 | 0.000848 | 0.279541 |
| Bronchus and lung | UK Biobank | Vitamin C | rs10136000 | -0.00068 | 0.000847 | 0.420769 |
| Bronchus and lung | UK Biobank | Vitamin C | rs117885456 | -0.00069 | 0.000855 | 0.421369 |
| Bronchus and lung | UK Biobank | Vitamin C | rs13028225 | -0.00093 | 0.000895 | 0.298825 |
| Bronchus and lung | UK Biobank | Vitamin C | rs174547 | -0.0006 | 0.000845 | 0.480713 |
| Bronchus and lung | UK Biobank | Vitamin C | rs2559850 | -0.00106 | 0.00087 | 0.22282 |
| Bronchus and lung | UK Biobank | Vitamin C | rs33972313 | -0.0013 | 0.001152 | 0.258781 |
| Bronchus and lung | UK Biobank | Vitamin C | rs56738967 | -0.00066 | 0.000849 | 0.439305 |
| Bronchus and lung | UK Biobank | Vitamin C | rs6693447 | -0.001 | 0.000849 | 0.240555 |
| Bronchus and lung | UK Biobank | Vitamin C | rs9895661 | -0.00062 | 0.000858 | 0.466935 |
| Bronchus and lung | UK Biobank | Vitamin C | All | -0.00082 | 0.000832 | 0.323455 |
| Bronchus and lung | FinnGen Biobank | Vitamin C | rs10051765 | -0.01854 | 0.608556 | 0.975699 |
| Bronchus and lung | FinnGen Biobank | Vitamin C | rs10136000 | 0.128119 | 0.586483 | 0.827076 |
| Bronchus and lung | FinnGen Biobank | Vitamin C | rs13028225 | 0.01866 | 0.675469 | 0.977961 |
| Bronchus and lung | FinnGen Biobank | Vitamin C | rs174547 | 0.318186 | 0.412226 | 0.44019 |
| Bronchus and lung | FinnGen Biobank | Vitamin C | rs33972313 | -0.51768 | 0.774853 | 0.504073 |
| Bronchus and lung | FinnGen Biobank | Vitamin C | rs56738967 | 0.027491 | 0.616028 | 0.964405 |
| Bronchus and lung | FinnGen Biobank | Vitamin C | rs6693447 | 0.014682 | 0.616216 | 0.980991 |
| Bronchus and lung | FinnGen Biobank | Vitamin C | All | 0.033919 | 0.546012 | 0.950467 |
| Lung | ILCCO | Vitamin C | rs10051765 | 0.04384 | 0.206599 | 0.831954 |
| Lung | ILCCO | Vitamin C | rs10136000 | -0.00417 | 0.209862 | 0.984139 |
| Lung | ILCCO | Vitamin C | rs13028225 | 0.033497 | 0.228453 | 0.883427 |
| Lung | ILCCO | Vitamin C | rs174547 | 0.090565 | 0.16338 | 0.57936 |
| Lung | ILCCO | Vitamin C | rs2559850 | -0.00759 | 0.220699 | 0.972576 |
| Lung | ILCCO | Vitamin C | rs33972313 | -0.03745 | 0.283404 | 0.894858 |
| Lung | ILCCO | Vitamin C | rs56738967 | 0.00387 | 0.214614 | 0.985612 |
| Lung | ILCCO | Vitamin C | rs6693447 | 0.060164 | 0.19716 | 0.760249 |
| Lung | ILCCO | Vitamin C | rs9895661 | -0.08147 | 0.177597 | 0.646441 |
| Lung | ILCCO | Vitamin C | All | 0.014008 | 0.196618 | 0.943202 |
| Breast | UK Biobank | Vitamin C | rs10051765 | 0.002577 | 0.001625 | 0.112684 |
| Breast | UK Biobank | Vitamin C | rs10136000 | 0.002084 | 0.001623 | 0.199168 |
| Breast | UK Biobank | Vitamin C | rs117885456 | 0.002679 | 0.001638 | 0.101918 |
| Breast | UK Biobank | Vitamin C | rs13028225 | 0.002653 | 0.001716 | 0.12219 |
| Breast | UK Biobank | Vitamin C | rs174547 | 0.002163 | 0.001621 | 0.182001 |
| Breast | UK Biobank | Vitamin C | rs2559850 | 0.00242 | 0.001669 | 0.147134 |
| Breast | UK Biobank | Vitamin C | rs33972313 | 0.001194 | 0.002199 | 0.587087 |
| Breast | UK Biobank | Vitamin C | rs56738967 | 0.002538 | 0.001627 | 0.118739 |
| Breast | UK Biobank | Vitamin C | rs6693447 | 0.001753 | 0.001628 | 0.281709 |
| Breast | UK Biobank | Vitamin C | rs9895661 | 0.002461 | 0.001646 | 0.134913 |
| Breast | UK Biobank | Vitamin C | All | 0.002297 | 0.001594 | 0.149642 |
| Breast | FinnGen Biobank | Vitamin C | rs10051765 | -0.21168 | 0.326806 | 0.517156 |
| Breast | FinnGen Biobank | Vitamin C | rs10136000 | -0.22259 | 0.31888 | 0.485158 |
| Breast | FinnGen Biobank | Vitamin C | rs13028225 | -0.13901 | 0.364602 | 0.703006 |
| Breast | FinnGen Biobank | Vitamin C | rs174547 | -0.17319 | 0.333657 | 0.603708 |
| Breast | FinnGen Biobank | Vitamin C | rs33972313 | 0.183543 | 0.410578 | 0.65485 |
| Breast | FinnGen Biobank | Vitamin C | rs56738967 | -0.13286 | 0.326834 | 0.684378 |
| Breast | FinnGen Biobank | Vitamin C | rs6693447 | -0.3309 | 0.192855 | 0.0862 |
| Breast | FinnGen Biobank | Vitamin C | All | -0.17204 | 0.296927 | 0.562316 |
| Breast | BCAC | Vitamin C | rs10051765 | 0.040788 | 0.064832 | 0.529257 |
| Breast | BCAC | Vitamin C | rs10136000 | 0.031922 | 0.060307 | 0.59658 |
| Breast | BCAC | Vitamin C | rs13028225 | 0.062613 | 0.068236 | 0.358832 |
| Breast | BCAC | Vitamin C | rs174547 | 0.055451 | 0.062186 | 0.372553 |
| Breast | BCAC | Vitamin C | rs33972313 | 0.007953 | 0.09121 | 0.930513 |
| Breast | BCAC | Vitamin C | rs56738967 | 0.048687 | 0.065569 | 0.457766 |
| Breast | BCAC | Vitamin C | rs6693447 | 0.024562 | 0.054312 | 0.651096 |
| Breast | BCAC | Vitamin C | rs9895661 | 0.073795 | 0.052866 | 0.16275 |
| Breast | BCAC | Vitamin C | All | 0.045177 | 0.059461 | 0.44739 |
| Pancrease | PanScan1 | Vitamin C | rs10136000 | 0.278627 | 0.523029 | 0.594229 |
| Pancrease | PanScan1 | Vitamin C | rs13028225 | 0.51744 | 0.685444 | 0.45031 |
| Pancrease | PanScan1 | Vitamin C | rs174547 | 0.227031 | 0.51804 | 0.661205 |
| Pancrease | PanScan1 | Vitamin C | rs9895661 | 0.521743 | 0.557997 | 0.349773 |
| Pancrease | PanScan1 | Vitamin C | All | 0.364845 | 0.485607 | 0.452462 |
| Pancrease | FinnGen Biobank | Vitamin C | rs10051765 | 0.044097 | 0.611749 | 0.942536 |
| Pancrease | FinnGen Biobank | Vitamin C | rs10136000 | -0.24217 | 0.704203 | 0.730932 |
| Pancrease | FinnGen Biobank | Vitamin C | rs13028225 | -0.46265 | 0.743713 | 0.533893 |
| Pancrease | FinnGen Biobank | Vitamin C | rs174547 | -0.43977 | 0.609439 | 0.47054 |
| Pancrease | FinnGen Biobank | Vitamin C | rs33972313 | -0.11459 | 0.976168 | 0.906555 |
| Pancrease | FinnGen Biobank | Vitamin C | rs56738967 | -0.32063 | 0.691352 | 0.642809 |
| Pancrease | FinnGen Biobank | Vitamin C | rs6693447 | -0.1454 | 0.683853 | 0.831626 |
| Pancrease | FinnGen Biobank | Vitamin C | All | -0.24413 | 0.625959 | 0.696531 |
| Colon | UK Biobank | Vitamin C | rs10051765 | -0.00276 | 0.001272 | 0.029876 |
| Colon | UK Biobank | Vitamin C | rs10136000 | -0.0034 | 0.001267 | 0.00736 |
| Colon | UK Biobank | Vitamin C | rs174547 | -0.00346 | 0.001259 | 0.005931 |
| Colon | UK Biobank | Vitamin C | rs2559850 | -0.0038 | 0.001434 | 0.008091 |
| Colon | UK Biobank | Vitamin C | rs56738967 | -0.0039 | 0.001279 | 0.002303 |
| Colon | UK Biobank | Vitamin C | rs6693447 | -0.00354 | 0.001284 | 0.005873 |
| Colon | UK Biobank | Vitamin C | All | -0.00346 | 0.001183 | 0.003405 |
| Colon | FinnGen Biobank | Vitamin C | rs10051765 | -0.34366 | 0.419368 | 0.412521 |
| Colon | FinnGen Biobank | Vitamin C | rs10136000 | -0.3544 | 0.423129 | 0.402273 |
| Colon | FinnGen Biobank | Vitamin C | rs13028225 | -0.37454 | 0.52129 | 0.472453 |
| Colon | FinnGen Biobank | Vitamin C | rs174547 | -0.60643 | 0.399248 | 0.128782 |
| Colon | FinnGen Biobank | Vitamin C | rs33972313 | -0.55305 | 0.668701 | 0.408212 |
| Colon | FinnGen Biobank | Vitamin C | rs56738967 | -0.595 | 0.424433 | 0.16095 |
| Colon | FinnGen Biobank | Vitamin C | rs6693447 | -0.49826 | 0.481518 | 0.300772 |
| Colon | FinnGen Biobank | Vitamin C | All | -0.47184 | 0.42845 | 0.270775 |
| Rectum | UK Biobank | Vitamin C | rs10051765 | -0.00184 | 0.001262 | 0.143833 |
| Rectum | UK Biobank | Vitamin C | rs10136000 | -0.00226 | 0.001257 | 0.072154 |
| Rectum | UK Biobank | Vitamin C | rs174547 | -0.00137 | 0.001249 | 0.273788 |
| Rectum | UK Biobank | Vitamin C | rs2559850 | -0.0008 | 0.001422 | 0.575144 |
| Rectum | UK Biobank | Vitamin C | rs56738967 | -0.00155 | 0.001269 | 0.221739 |
| Rectum | UK Biobank | Vitamin C | rs6693447 | -0.0018 | 0.001273 | 0.156837 |
| Rectum | UK Biobank | Vitamin C | All | -0.00163 | 0.001173 | 0.163891 |
| Rectum | FinnGen Biobank | Vitamin C | rs10051765 | -0.15065 | 0.576642 | 0.793892 |
| Rectum | FinnGen Biobank | Vitamin C | rs10136000 | -0.13443 | 0.574844 | 0.815101 |
| Rectum | FinnGen Biobank | Vitamin C | rs13028225 | -0.31277 | 0.631441 | 0.620367 |
| Rectum | FinnGen Biobank | Vitamin C | rs174547 | -0.12227 | 0.574324 | 0.831408 |
| Rectum | FinnGen Biobank | Vitamin C | rs33972313 | -0.38339 | 0.79588 | 0.63001 |
| Rectum | FinnGen Biobank | Vitamin C | rs56738967 | -0.13064 | 0.576469 | 0.820724 |
| Rectum | FinnGen Biobank | Vitamin C | rs6693447 | -0.17468 | 0.577376 | 0.762246 |
| Rectum | FinnGen Biobank | Vitamin C | All | -0.18461 | 0.559586 | 0.741469 |
| Kidney | UK Biobank | Vitamin C | rs10051765 | 0.000809 | 0.001191 | 0.497097 |
| Kidney | UK Biobank | Vitamin C | rs174547 | 0.001476 | 0.001176 | 0.209406 |
| Kidney | UK Biobank | Vitamin C | rs2559850 | 0.000218 | 0.001375 | 0.87397 |
| Kidney | UK Biobank | Vitamin C | rs56738967 | 0.001041 | 0.001199 | 0.385524 |
| Kidney | UK Biobank | Vitamin C | rs6693447 | 0.001381 | 0.001204 | 0.251415 |
| Kidney | UK Biobank | Vitamin C | All | 0.001026 | 0.001094 | 0.348315 |
| Kidney | FinnGen Biobank | Vitamin C | rs10051765 | 0.036197 | 0.79135 | 0.963517 |
| Kidney | FinnGen Biobank | Vitamin C | rs10136000 | 0.166446 | 0.735319 | 0.820922 |
| Kidney | FinnGen Biobank | Vitamin C | rs13028225 | -0.26006 | 0.823605 | 0.752187 |
| Kidney | FinnGen Biobank | Vitamin C | rs174547 | 0.126714 | 0.759033 | 0.867416 |
| Kidney | FinnGen Biobank | Vitamin C | rs33972313 | -0.57332 | 1.031738 | 0.578428 |
| Kidney | FinnGen Biobank | Vitamin C | rs56738967 | -0.06855 | 0.774749 | 0.929499 |
| Kidney | FinnGen Biobank | Vitamin C | rs6693447 | 0.374145 | 0.53713 | 0.486076 |
| Kidney | FinnGen Biobank | Vitamin C | All | 0.018808 | 0.701758 | 0.978618 |
| Bladder | UK Biobank | Vitamin C | rs10051765 | -0.00067 | 0.001223 | 0.585194 |
| Bladder | UK Biobank | Vitamin C | rs174547 | -0.00082 | 0.00117 | 0.485424 |
| Bladder | UK Biobank | Vitamin C | rs2559850 | -0.00163 | 0.001361 | 0.23163 |
| Bladder | UK Biobank | Vitamin C | rs56738967 | 0.000108 | 0.001192 | 0.927496 |
| Bladder | UK Biobank | Vitamin C | rs6693447 | -0.00032 | 0.001192 | 0.789047 |
| Bladder | UK Biobank | Vitamin C | All | -0.00062 | 0.001084 | 0.567694 |
| Bladder | FinnGen Biobank | Vitamin C | rs10051765 | 0.220023 | 0.749428 | 0.769073 |
| Bladder | FinnGen Biobank | Vitamin C | rs10136000 | 0.424382 | 0.553776 | 0.443472 |
| Bladder | FinnGen Biobank | Vitamin C | rs13028225 | 0.323911 | 0.816816 | 0.691697 |
| Bladder | FinnGen Biobank | Vitamin C | rs174547 | 0.362728 | 0.639188 | 0.570386 |
| Bladder | FinnGen Biobank | Vitamin C | rs33972313 | -0.72332 | 0.884992 | 0.413747 |
| Bladder | FinnGen Biobank | Vitamin C | rs56738967 | 0.093226 | 0.745394 | 0.900469 |
| Bladder | FinnGen Biobank | Vitamin C | rs6693447 | 0.041126 | 0.724999 | 0.954763 |
| Bladder | FinnGen Biobank | Vitamin C | All | 0.162815 | 0.670993 | 0.808278 |
| Prostate | UK Biobank | Vitamin C | rs10051765 | 0.000757 | 0.001994 | 0.70412 |
| Prostate | UK Biobank | Vitamin C | rs10136000 | -0.00018 | 0.00199 | 0.927537 |
| Prostate | UK Biobank | Vitamin C | rs117885456 | -0.0003 | 0.002026 | 0.880782 |
| Prostate | UK Biobank | Vitamin C | rs13028225 | 0.00028 | 0.002236 | 0.900405 |
| Prostate | UK Biobank | Vitamin C | rs174547 | 0.000134 | 0.001984 | 0.946244 |
| Prostate | UK Biobank | Vitamin C | rs2559850 | -0.00044 | 0.002106 | 0.835834 |
| Prostate | UK Biobank | Vitamin C | rs56738967 | 5.19E-05 | 0.002 | 0.979282 |
| Prostate | UK Biobank | Vitamin C | rs6693447 | -0.00035 | 0.002003 | 0.861185 |
| Prostate | UK Biobank | Vitamin C | rs9895661 | 0.0008 | 0.002047 | 0.695849 |
| Prostate | UK Biobank | Vitamin C | All | 8.27E-05 | 0.001922 | 0.965701 |
| Prostate | FinnGen Biobank | Vitamin C | rs10051765 | 0.371471 | 0.229836 | 0.106041 |
| Prostate | FinnGen Biobank | Vitamin C | rs10136000 | 0.333751 | 0.229179 | 0.145311 |
| Prostate | FinnGen Biobank | Vitamin C | rs13028225 | 0.280999 | 0.251771 | 0.264383 |
| Prostate | FinnGen Biobank | Vitamin C | rs174547 | 0.39343 | 0.228968 | 0.085747 |
| Prostate | FinnGen Biobank | Vitamin C | rs33972313 | 0.343242 | 0.317441 | 0.279573 |
| Prostate | FinnGen Biobank | Vitamin C | rs56738967 | 0.336538 | 0.22982 | 0.143096 |
| Prostate | FinnGen Biobank | Vitamin C | rs6693447 | 0.255597 | 0.230201 | 0.26686 |
| Prostate | FinnGen Biobank | Vitamin C | All | 0.331199 | 0.223098 | 0.137665 |
| Prostate | PRACTICAL | Vitamin C | rs10051765 | -0.04155 | 0.045099 | 0.356949 |
| Prostate | PRACTICAL | Vitamin C | rs10136000 | -0.03699 | 0.045124 | 0.412334 |
| Prostate | PRACTICAL | Vitamin C | rs117885456 | -0.02785 | 0.045223 | 0.537952 |
| Prostate | PRACTICAL | Vitamin C | rs13028225 | -0.04824 | 0.047794 | 0.312811 |
| Prostate | PRACTICAL | Vitamin C | rs174547 | -0.03264 | 0.04508 | 0.469052 |
| Prostate | PRACTICAL | Vitamin C | rs2559850 | -0.04346 | 0.046295 | 0.347867 |
| Prostate | PRACTICAL | Vitamin C | rs33972313 | -0.03819 | 0.060944 | 0.530886 |
| Prostate | PRACTICAL | Vitamin C | rs56738967 | -0.02889 | 0.045276 | 0.523409 |
| Prostate | PRACTICAL | Vitamin C | rs6693447 | -0.03238 | 0.045223 | 0.473993 |
| Prostate | PRACTICAL | Vitamin C | rs9895661 | -0.01613 | 0.045835 | 0.724974 |
| Prostate | PRACTICAL | Vitamin C | All | -0.03432 | 0.04428 | 0.438245 |
| Ovary | UK Biobank | Vitamin C | rs10051765 | -0.00238 | 0.001171 | 0.041773 |
| Ovary | UK Biobank | Vitamin C | rs174547 | -0.00234 | 0.001157 | 0.042766 |
| Ovary | UK Biobank | Vitamin C | rs2559850 | -0.00199 | 0.001351 | 0.141499 |
| Ovary | UK Biobank | Vitamin C | rs56738967 | -0.0021 | 0.001184 | 0.075878 |
| Ovary | UK Biobank | Vitamin C | rs6693447 | -0.00219 | 0.001184 | 0.064441 |
| Ovary | UK Biobank | Vitamin C | All | -0.00221 | 0.001077 | 0.039778 |
| Ovary | FinnGen Biobank | Vitamin C | rs10051765 | -0.06373 | 0.684847 | 0.925862 |
| Ovary | FinnGen Biobank | Vitamin C | rs10136000 | -0.08062 | 0.682835 | 0.90601 |
| Ovary | FinnGen Biobank | Vitamin C | rs13028225 | 0.17102 | 0.749782 | 0.819574 |
| Ovary | FinnGen Biobank | Vitamin C | rs174547 | -0.09913 | 0.682067 | 0.884443 |
| Ovary | FinnGen Biobank | Vitamin C | rs33972313 | 0.25959 | 0.944758 | 0.783494 |
| Ovary | FinnGen Biobank | Vitamin C | rs56738967 | 0.036376 | 0.684698 | 0.957631 |
| Ovary | FinnGen Biobank | Vitamin C | rs6693447 | -0.35386 | 0.685704 | 0.605815 |
| Ovary | FinnGen Biobank | Vitamin C | All | -0.0443 | 0.664568 | 0.946853 |
| Ovary | OCAC | Vitamin C | rs10051765 | -0.08381 | 0.087452 | 0.337906 |
| Ovary | OCAC | Vitamin C | rs10136000 | -0.08495 | 0.086742 | 0.327421 |
| Ovary | OCAC | Vitamin C | rs13028225 | -0.07748 | 0.095601 | 0.417695 |
| Ovary | OCAC | Vitamin C | rs174547 | -0.10927 | 0.079821 | 0.171033 |
| Ovary | OCAC | Vitamin C | rs33972313 | 0.043907 | 0.113846 | 0.699741 |
| Ovary | OCAC | Vitamin C | rs56738967 | -0.05733 | 0.084068 | 0.49531 |
| Ovary | OCAC | Vitamin C | rs6693447 | -0.08327 | 0.087934 | 0.343676 |
| Ovary | OCAC | Vitamin C | rs9895661 | -0.08321 | 0.090148 | 0.356012 |
| Ovary | OCAC | Vitamin C | All | -0.07435 | 0.08085 | 0.357806 |
| Uterus/endometrium | UK Biobank | Vitamin C | rs10051765 | -0.00024 | 0.00121 | 0.843561 |
| Uterus/endometrium | UK Biobank | Vitamin C | rs174547 | 9.64E-05 | 0.001195 | 0.935733 |
| Uterus/endometrium | UK Biobank | Vitamin C | rs2559850 | -0.0008 | 0.001398 | 0.567726 |
| Uterus/endometrium | UK Biobank | Vitamin C | rs56738967 | -0.00038 | 0.001218 | 0.752623 |
| Uterus/endometrium | UK Biobank | Vitamin C | rs6693447 | -0.00016 | 0.001223 | 0.895183 |
| Uterus/endometrium | UK Biobank | Vitamin C | All | -0.00027 | 0.001111 | 0.808764 |
| Corpus uteri | FinnGen Biobank | Vitamin C | rs10051765 | 0.306725 | 0.486079 | 0.528028 |
| Corpus uteri | FinnGen Biobank | Vitamin C | rs10136000 | 0.291164 | 0.484673 | 0.548011 |
| Corpus uteri | FinnGen Biobank | Vitamin C | rs13028225 | 0.064814 | 0.531629 | 0.902966 |
| Corpus uteri | FinnGen Biobank | Vitamin C | rs174547 | 0.231512 | 0.48423 | 0.632576 |
| Corpus uteri | FinnGen Biobank | Vitamin C | rs33972313 | -0.34134 | 0.674632 | 0.612886 |
| Corpus uteri | FinnGen Biobank | Vitamin C | rs56738967 | 0.368825 | 0.485982 | 0.447896 |
| Corpus uteri | FinnGen Biobank | Vitamin C | rs6693447 | 0.238643 | 0.486679 | 0.623886 |
| Corpus uteri | FinnGen Biobank | Vitamin C | All | 0.206844 | 0.47188 | 0.661139 |

Abbreviations: ILCCO, International Lung Cancer Consortium; BCAC, Breast Cancer Association Consortium; PanScan1, Pancreatic Cancer Cohort Consortium GWAS; PRACTICAL, Prostate Cancer Association group To Investigate Cancer Associated Alterations in the Genome; OCAC, Ovarian Cancer Association Consortium.
