# Supplementary figures and images for: The Association Between Vitamin C and Cancer: A Two-Sample Mendelian Randomization Study
Source: Front Genet. 2022 May 5;13:868408. doi: 10.3389/fgene.2022.868408 (PMC9117647; doi:10.3389/fgene.2022.868408)

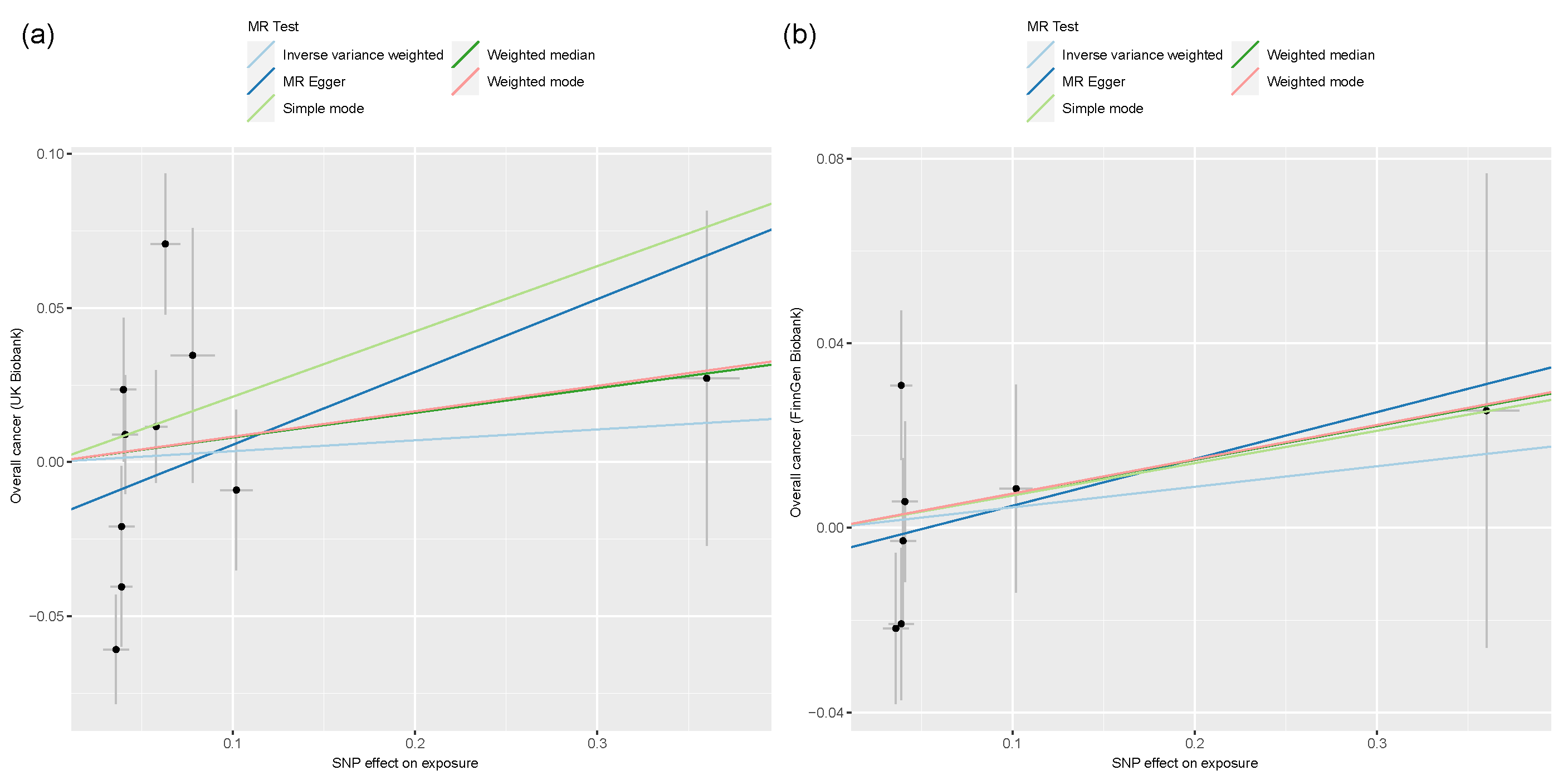

Supplement: Supplementary file 1 [file DataSheet1.ZIP › Supplementary Figure 1 overall.tiff]

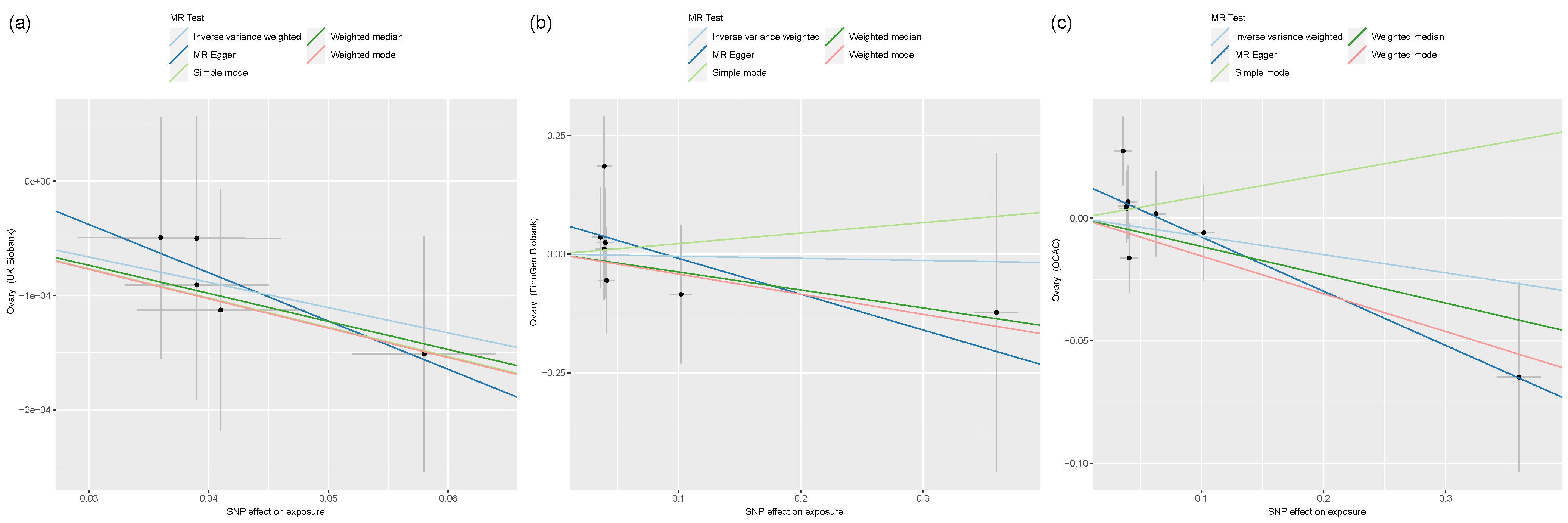

Supplement: Supplementary file 1 [file DataSheet1.ZIP › Supplementary Figure 10 ovary.tif]

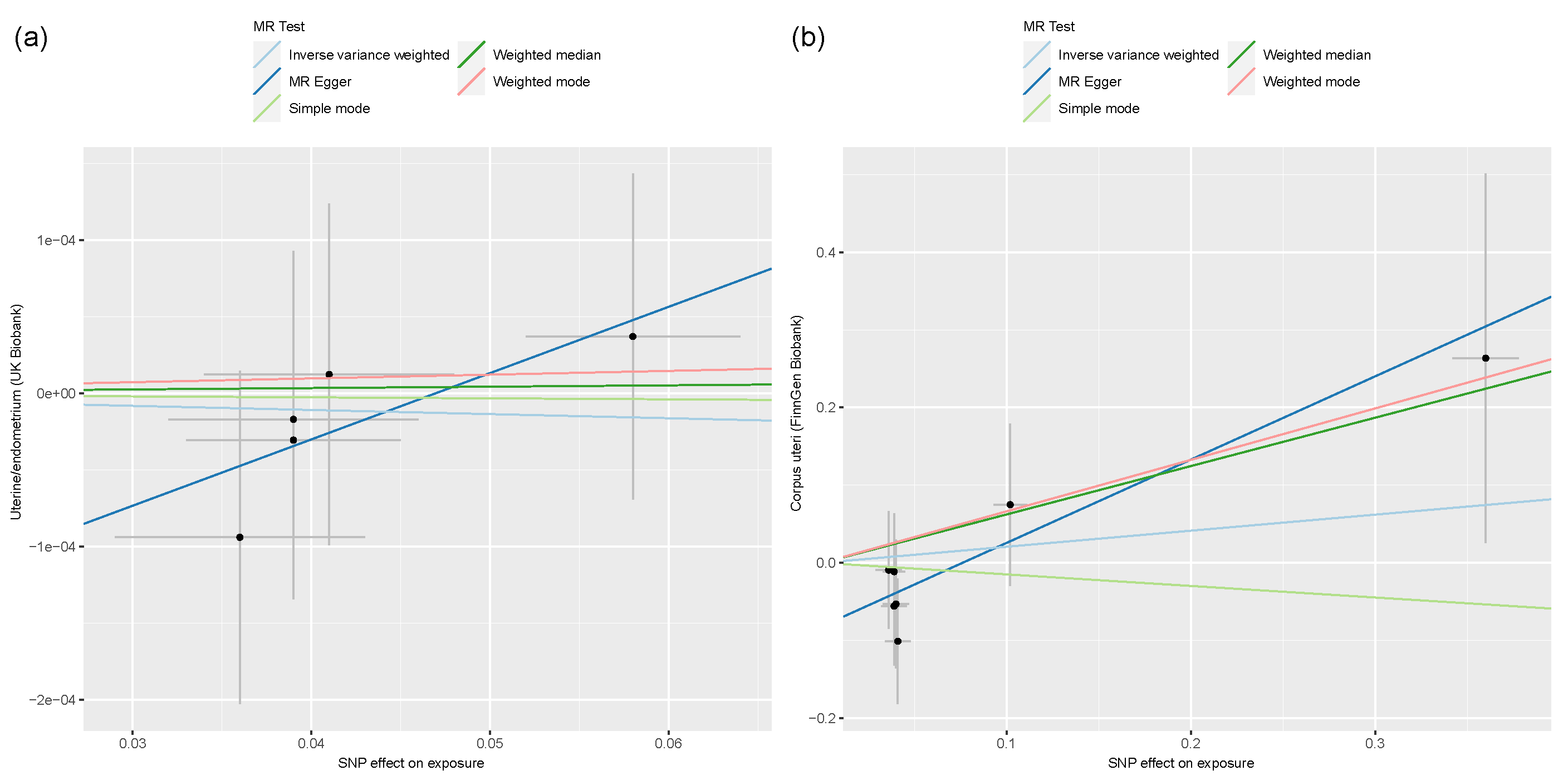

Supplement: Supplementary file 1 [file DataSheet1.ZIP › Supplementary Figure 11 uterine.tif]

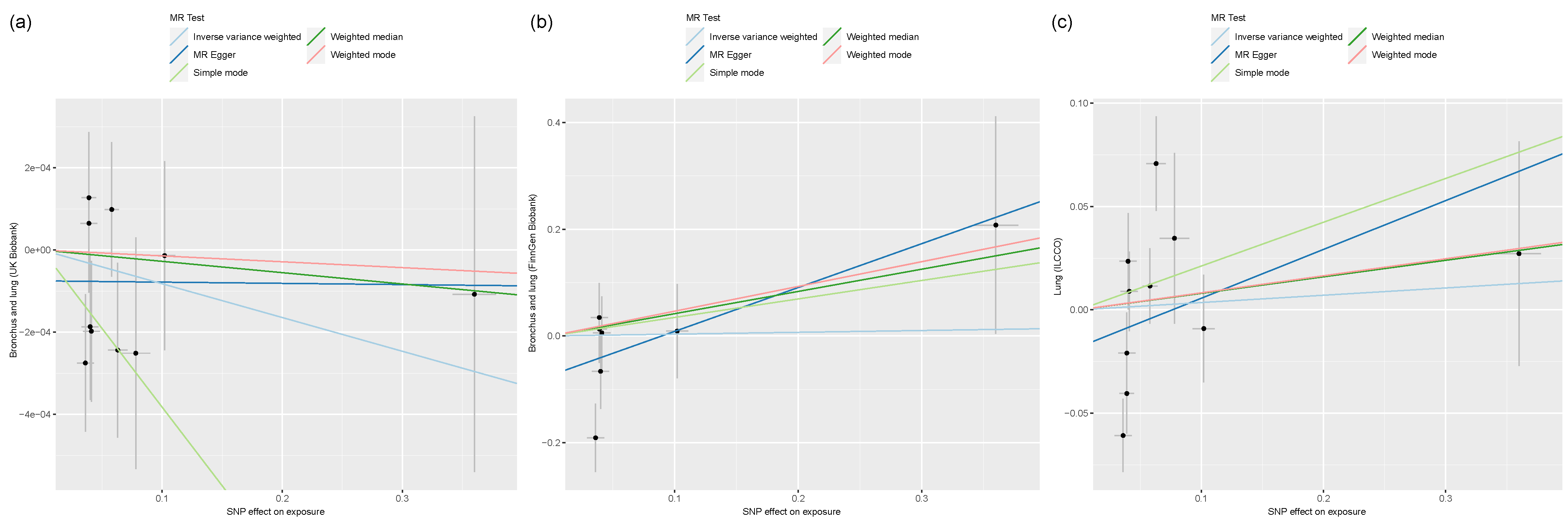

Supplement: Supplementary file 1 [file DataSheet1.ZIP › Supplementary Figure 2 lung.tif]

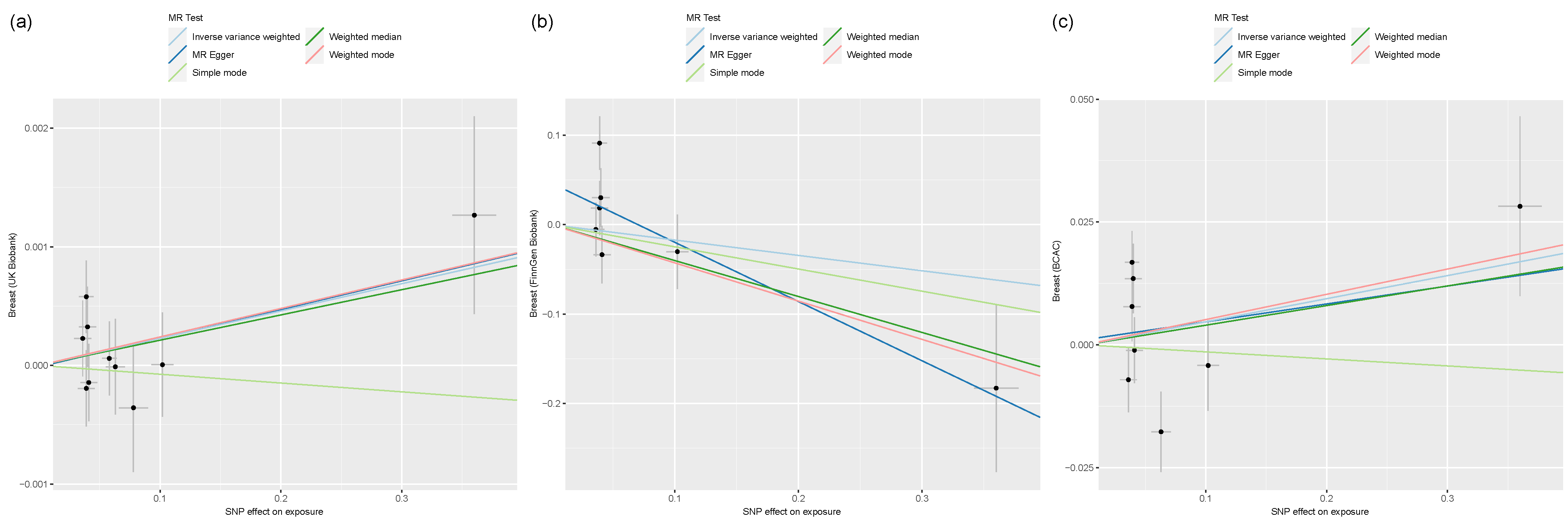

Supplement: Supplementary file 1 [file DataSheet1.ZIP › Supplementary Figure 3 breast.tif]

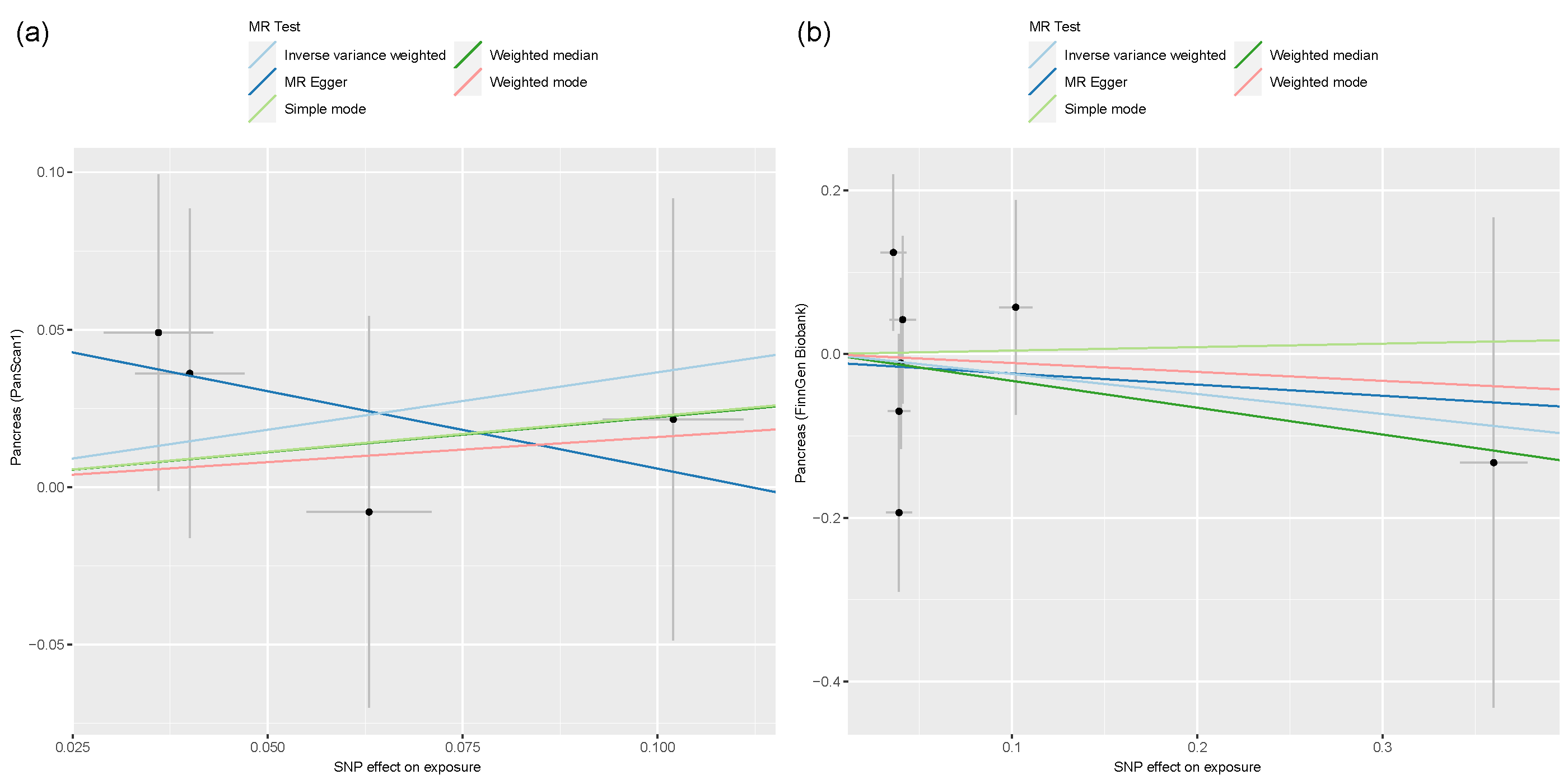

Supplement: Supplementary file 1 [file DataSheet1.ZIP › Supplementary Figure 4 pancreas.tif]

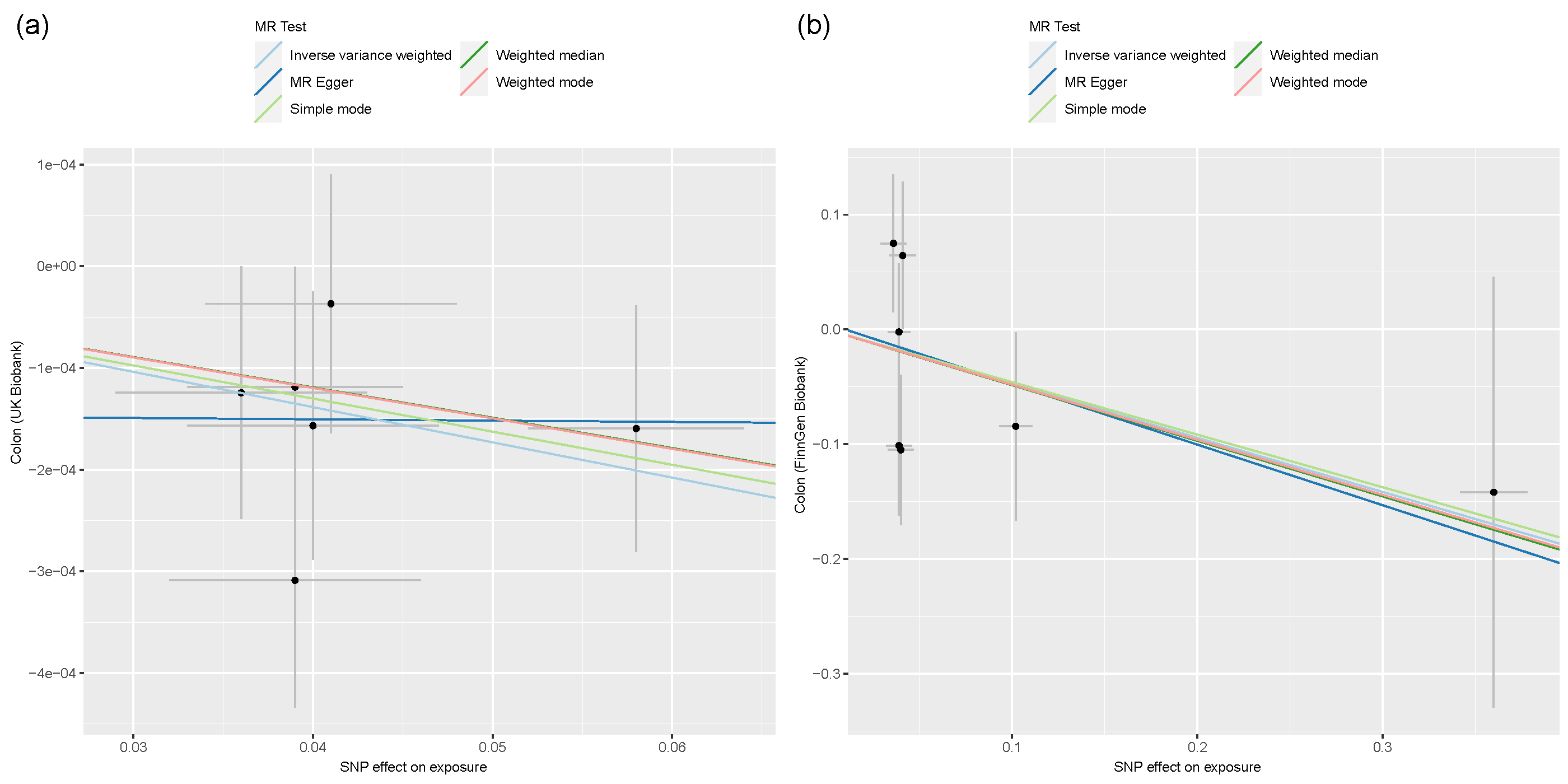

Supplement: Supplementary file 1 [file DataSheet1.ZIP › Supplementary Figure 5 colon.tif]

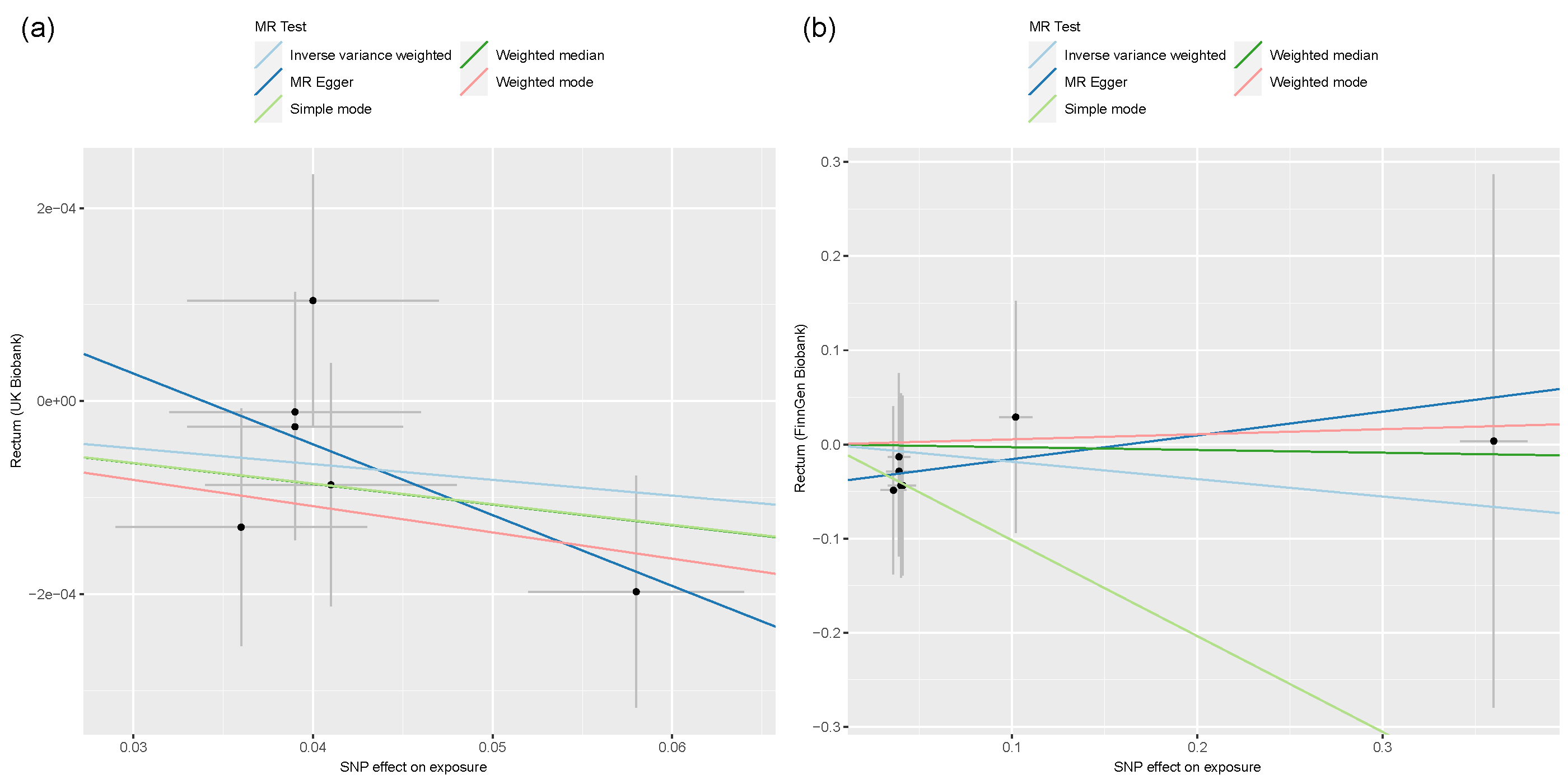

Supplement: Supplementary file 1 [file DataSheet1.ZIP › Supplementary Figure 6 rectum.tif]

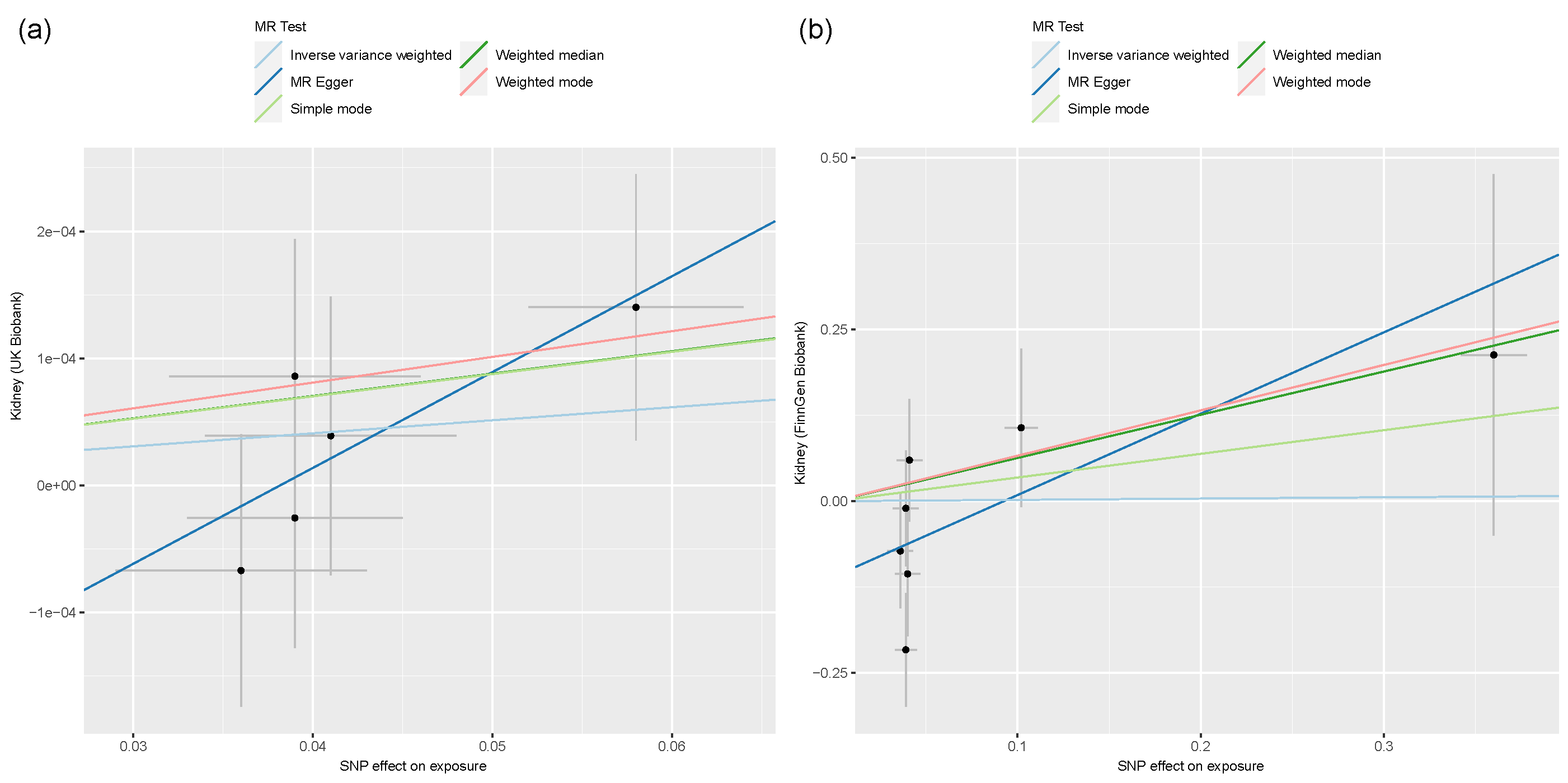

Supplement: Supplementary file 1 [file DataSheet1.ZIP › Supplementary Figure 7 kidney.tif]

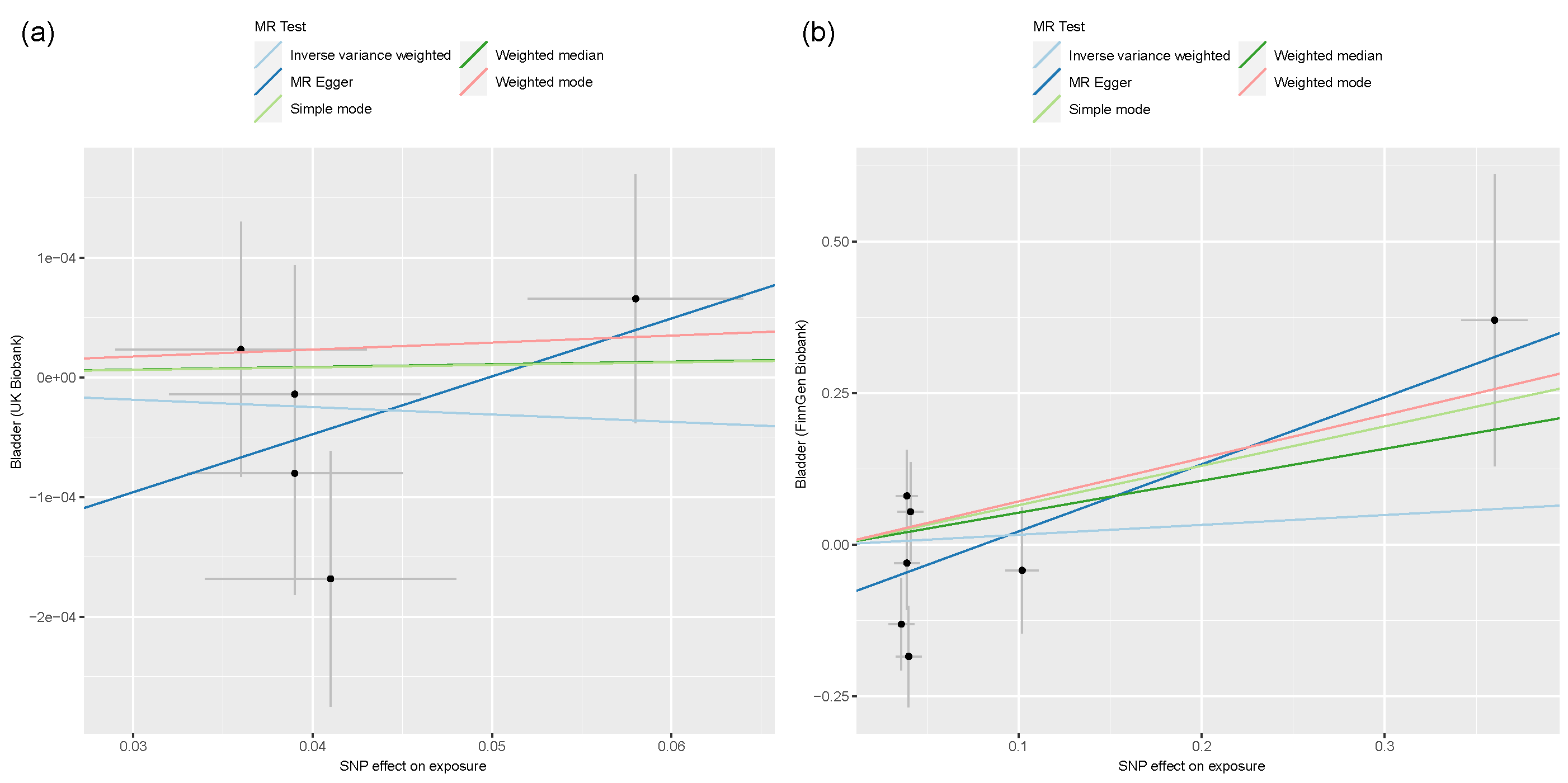

Supplement: Supplementary file 1 [file DataSheet1.ZIP › Supplementary Figure 8 bladder.tif]

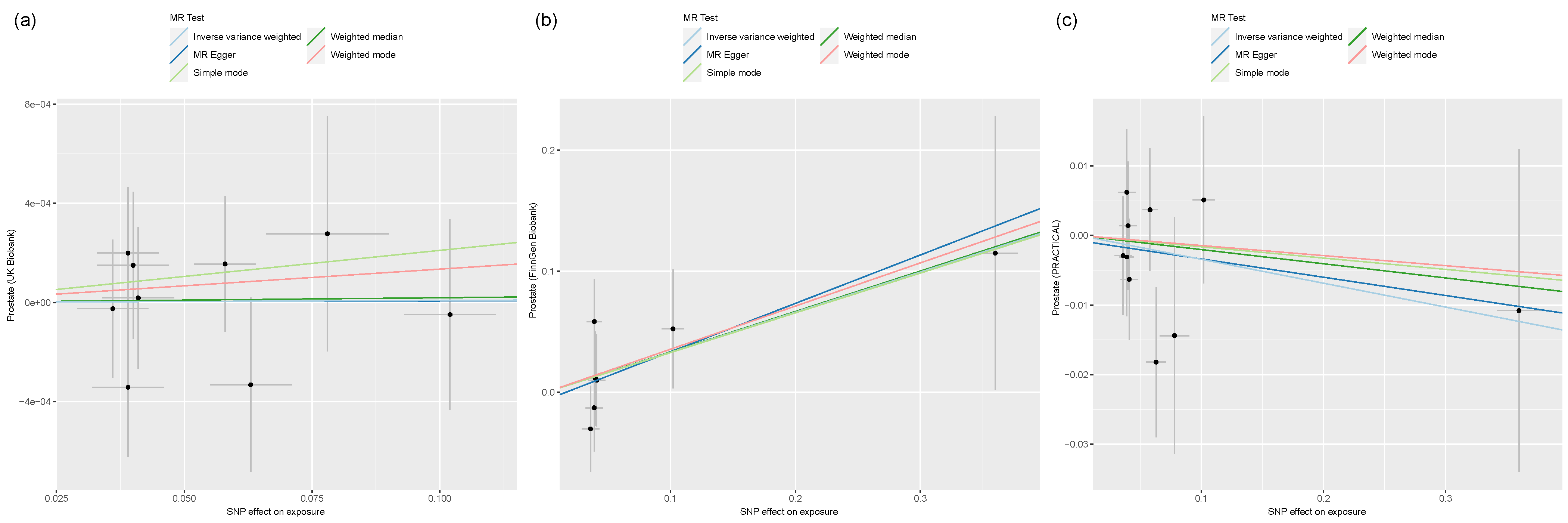

Supplement: Supplementary file 1 [file DataSheet1.ZIP › Supplementary Figure 9 prostate.tif]
